# FcRn Expression in Wildtype Mice, Transgenic Mice, and in Human Tissues

**DOI:** 10.3390/biom8040115

**Published:** 2018-10-15

**Authors:** Tommy Li, Joseph P. Balthasar

**Affiliations:** Department of Pharmaceutical Sciences, School of Pharmacy and Pharmaceutical Sciences, University at Buffalo, Buffalo, NY 14214, USA; tommyli@buffalo.edu

**Keywords:** FcRn, human FcRn transgenic mouse, mRNA, protein, tissue expression

## Abstract

Quantitative real-time PCR and Western blot methods were developed to assess neonatal Fc-receptor (FcRn) mRNA and protein expression in human FcRn transgenic mice, Swiss Webster mice, and in select human tissues. Additionally, FcRn turnover was evaluated via pulse-chase. FcRn mRNA expression was significantly higher in transgenic mice when compared to mouse FcRn mRNA in Swiss Webster mice and it ranged from 184-fold higher in the kidney to 109,000-fold higher in the skin. FcRn protein expression was found to be 13-fold lower in kidney to 5.6-fold higher in lung obtained from transgenic mice compared to FcRn protein expression in lung samples obtained from Swiss Webster mice. FcRn protein expression in human liver and small intestine tissues matched more closely with FcRn expression in Swiss Webster mice but were significantly lower when compared to values found from Swiss Webster and transgenic mice. Although FcRn mRNA expression correlated significantly with protein expression (*p* < 0.0005), the correlation coefficient was only 0.113. As such, the measurement of FcRn protein may be preferred to FcRn mRNA for quantitative applications. Significant differences were found in FcRn expression in transgenic mice, Swiss Webster mice, and human tissues, which may have implications for the use of mouse models in the assessment of monoclonal antibody disposition, efficacy, and safety.

## 1. Introduction

Interest in the development of therapeutic monoclonal antibody (mAb) drugs has grown rapidly in the past few decades. The first mAb approved for therapeutic use in the United States, Muromonab-CD3, exhibited rapid elimination [1]. It is now appreciated that the rapid clearance of Muromonab-CD3, which is a murine immunoglobulin G (IgG)2a mAb, is partially explained by the poor binding of mouse antibodies to the human neonatal Fc receptor (hFcRn), which protects IgG antibodies from catabolism [2]. The importance of FcRn as a determinant of mAb pharmacokinetics and pharmacodynamics (PK/PD) has led to several publications focusing on the species selectivity of FcRn-IgG interaction and the development of engineered mAb with improved hFcRn binding [3,4,5,6,7,8]. The finding of important between species differences in FcRn—IgG binding has led to the development of transgenic mouse models that express human FcRn in tissues with the intent that these models would provide more relevant projections of the clinical PK/PD of human or humanized mAb [9,10].

In light of the important role played by FcRn in IgG pharmacokinetics and the common use of wild-type and transgenic animal models in the preclinical assessment of mAb PK/PD, it is somewhat surprising that there has been little investigation of the between-species and between-model differences in FcRn expression [11,12]. Additionally, there has been no report of the kinetics of FcRn production and turnover in vivo or in cells in culture. Although FcRn expression and turnover kinetics may be important determinants of PK/PD for most mAb drugs, FcRn expression and turnover are expected to be key determinants of the PK/PD of anti-FcRn mAb, which are currently under the development for the treatment of autoimmune and allo-immune conditions [5,13,14,15]. In this work, we compared the tissue expression level of FcRn in hFcRn transgenic mice, Swiss Webster mice, and select human tissues to assess possible differences in the tissue selectivity and the extent of FcRn expression. In addition, we investigated the turnover kinetics of hFcRn in a human umbilical vein endothelial cell line.

## 2. Materials and Methods

### 2.1. Animals and Human Tissue Samples

Mouse tissue samples were obtained from 11–13 week old B6.Cg-*Fcgrt*tm1Dcr Tg/Tg(*FCGRT*)276Dcr homozygous transgenic mice that express human FcRn (The Jackson Laboratory, Bar Harbor, ME, USA) and 11–13 week old Swiss Webster mice (Harlan, Indianapolis, IN, USA). Animals were housed and handled in accordance with the guidelines of the University at Buffalo Institutional Animal Care and Use Committee. Mouse tissue samples (liver, lung, spleen, small intestine, kidney, muscle, heart, and skin) were collected from the animals and immediately frozen in liquid nitrogen and stored at −80 °C until used (note: tissues were not perfused prior to collection). Additionally, adult human liver and small intestine tissues, which had been collected and frozen in liquid nitrogen less than 24 h post mortem, were obtained from the Cooperative Human Tissue Network. Human tissues were stored at −80 °C until used.

### 2.2. PCR Primers

Taqman gene expression assays for *FCGRT* (Assay ID Hs01108967_m1) and for *Fcgrt* (Assay ID Mm00438887_m1) were purchased from Invitrogen (Carlsbad, CA, USA). Matching Taqman gene expression assays for 18s (Assay ID Hs99999901_s1) and for Rn18s (Assay ID Mm03928990_g1) were purchased from Invitrogen.

### 2.3. Total RNA Isolation

Total RNA was isolated from 20 to 30 mg of tissue (liver, lung, spleen, small intestine, and kidney) obtained from the Swiss Webster mice and the transgenic mice using the RNeasy Mini Kit (Qiagen, Valencia, CA, USA). Similarly, total RNA was isolated from 20 to 30 mg of adult human liver and small intestine tissues using the RNeasy Mini Kit. For fibrous tissues (muscle, heart, and skin) of Swiss Webster and transgenic mice, total RNA was isolated from 20 to 30 mg of tissue using the RNeasy Fibrous Tissue Mini Kit (Qiagen). Tissues were homogenized in 600 µL of RLT buffer (Qiagen) containing 10% β-mercaptoethanol. For fibrous tissue, tissues were homogenized in 300 µL of RLT buffer containing 10% β-mercaptoethanol and 590 µL RNase free water was added to the homogenate along with 10 µL of proteinase K solution. The fibrous tissue homogenate was incubated at 55 °C for 10 min. Tissue homogenates were centrifuged at 10,000 relative centrifugal force (RCF) for 3 min. Supernatant was collected in a new micro centrifuge tube. One volume of 70% ethanol was added to supernatant collected from non-fibrous tissue homogenate. For fibrous tissue, 0.5 volume of 100% ethanol was added to the collected supernatant. The mixture was vortexed and 700 µL was transferred into the RNeasy spin column provided by the kit. The column was centrifuged at 10,000 RCF for 30 s and flow through was discarded. The non-fibrous tissue spin column was washed with 700 µL of RW1 buffer (Qiagen). For fibrous tissue, 350 µL of RW1 buffer was used to wash the column. The column was centrifuged at 10,000 RCF for 30 s and flow through was discarded. For fibrous tissue, 80 µL of the mixture contain 10 µL of DNase I stock solution and 70 µL buffer RDD (Qiagen) was added directly to the spin column and incubated at room temperature for 15 min. After the incubation, 350 µL of RW1 buffer was added to the fibrous tissue column and the column was centrifuged at 10,000 RCF for 30 s. Additionally, 500 µL of RPE buffer (Qiagen) was added to both non-fibrous and fibrous tissue columns and centrifuged at 10,000 RCF for 30 s. This wash step was repeated once more, and the column was centrifuged at 10,000 RCF for 2 min. RNA was eluded from the spin column using 30 µL of RNase free water at 10,000 RCF for 1 min. The concentration of extracted RNA was determined by measuring absorbance at 260 nm using the Nanodrop 2000 (Thermo Scientific, Wilmington, DE, USA). The purity of extracted RNA was determined by assessing the absorbance ratio A260/A280 and A260/A230. Extracted RNA samples from all tissues have A260/A280 > 1.8 and A260/A230 > 1.6. The integrity of extracted RNA was assessed by using gel electrophoresis and by resolving 5 µL of the extracted RNA on 1.2% agarose gel (Invitrogen) using Mini-Sub Cell GT Cell (Bio-Rad, Hercules, CA, USA) and following the established protocol [16].

### 2.4. Reverse Transcription of RNA to cDNA

Extracted RNA was converted to cDNA immediately after using the Superscript III Reverse Transcriptase Kit (Invitrogen). A total of 1000 ng of total RNA diluted to a final volume of 8 µL with diethylpyrocarbonate (DEPC)-treated water was mixed with 2 µL of the mixture containing an equal volume of 50 ng/µL random hexamers and 10 mM deoxyribonucleotide triphosphate (dNTP) mix. The mixture was vortexed and incubated at 65 °C for 50 min and then placed on ice for 1 min. The mixture was then mixed with 2 µL of 10 X reverse transcription (RT) buffer, 4 µL of 25 mM MgCl_2_, 2 µL of 0.1 M Dithiothreitol (DTT), 1 µL of RNaseOUT (40 U/µL), and 1 µL Superscript III Reverse Transcriptase (200 U/µL). All reagents are supplied within the Superscript III Reverse Transcriptase Kit (Invitrogen). The mixture was incubated at 25 °C for 10 min and then was incubated at 50 °C for 50 min. The reaction was terminated at 85 °C for 5 min and the samples were stored at −80 °C.

### 2.5. Production of mFcRn and hFcRn α-Chain cDNA Standards

The cDNA samples from liver tissue of transgenic mice and Swiss Webster mice were amplified with PCR. Additionally, 50 ng of cDNA was amplified in a total volume of 20 µL containing 56.25 nM of *FCGRT* or *Fcgrt* primer, 2.5 mM Mg^2+^ (New England BioLabs, Ipswich, MA, USA), 100 µM dNTP (New England BioLabs), and 0.5 IU Taq DNA polymerase (New England BioLabs) in PCR buffer (New England BioLabs). The PCR was performed on the Mastercycler gradient (Eppendorf, Hauppauge, NY, USA) with a thermal-cycling condition consisting of an initial denaturation at 50 °C for 10 min and 95 °C for 2 min, which was followed by 40 cycles composed of denaturation at 95 °C for 15 s and annealing at 60 °C for 60 s along with an extension at 70 °C for 30 s. A final extension step at 70 °C for 10 min was included at the end of the 40 cycles. The purity of the PCR product was assessed by using gel electrophoresis and by resolving 10 µL of the PCR product on 1.2% agarose gel (Invitrogen) utilizing Mini-Sub Cell GT Cell (Bio-Rad) following the established protocol [16]. The gel was visualized using the SYBR Gold nucleic acid gel stain (Invitrogen) and the purity of the PCR product was confirmed by the presence of a single discrete band at 107 base-pairs (bp) representing FcRn alpha chain PCR product. Mouse neonatal Fc receptor (mFcRn) and hFcRn PCR products were inserted into the plasmid vector using a TOPO TA Cloning Kit (Invitrogen). Additionally, 4 µL of the PCR product was mixed with 1 µL of salt solution and 1 µL of the TOPO vector provided by the kit. The mixture was incubated at room temperature for 5 min. After incubation, 2 µL of the mixture was added to a vial containing chemically competent *Escherichia coli* supplied by the kit. The cells were incubated on ice for 30 min and then heat shocked for 30 s at 42 °C. A total of 250 μL of pre-warmed S.O.C medium supplied by the kit was added to the cell and incubated in the shaker at 37 °C for 1 h. After the incubation, 25 µL of the culture was spread onto a pre-warmed Luria broth (LB) plate made from S-Gal/LB Agar Blend (Sigma-Aldrich, St. Louis, MO, USA) containing 100 μg/mL of ampicillin (Sigma-Aldrich). The LB plate was incubated overnight at 37 °C. Positive colonies were expanded in LB medium containing ampicillin overnight. Plasmid DNA was extracted from the expanded colony using Wizard Plus SV Minipreps DNA purification System (Promega, Madison, WI, USA). Additionally, 5 mL of the *E. coli* culture was centrifuged for 60 s at 16,000 RCF. The bacterial pellet was re-suspended in 250 µL of cell resuspension solution. Re-suspended bacteria were lysed by adding 250 µL of cell lysis solution. In addition, 10 µL of alkaline protease solution was added to the cell lysate and the cell lysate was incubated at room temperature for 5 min. Bacterial lysate was then neutralized with 350 µL of neutralization solution. The bacterial cell wall and precipitated protein was pelleted by centrifuging the lysate for 10 min at 16,000 RCF. Supernatant was transferred to the spin column and centrifuged for 1 min at 16,000 RCF. The spin column was washed twice with 750 µL and 250 µL of the wash solution. Extracted DNA was eluded from the spin column with 100 µL of nuclease free water. The concentration of extracted plasmid was determined using a Nanodrop 2000 (Thermo Scientific). The presence of the FcRn product in the cloned plasmid was confirmed with PCR using *Fcgrt* and *FCGRT* primer as well as sequencing of the cloned plasmid using primers provided by the TOPO TA cloning kit (Roswell Park DNA Sequencing Lab, Buffalo, NY, USA).

### 2.6. Real-Time PCR Analysis of FcRn cDNA

Quantification of FcRn cDNA in tissue samples was performed on a Mx3000P qPCR system (Agilent Technologies, Santa Clara, CA, USA). A total of 50 ng of cDNA was amplified in a volume of 20 µL containing 900 nM of *Fcgrt* primer or *FCGRT* primer along with matching 18s control primers in the TaqMan Universal PCR Master Mix (Invitrogen). The thermal-cycling condition consists of initial denaturation at 50 °C for 10 min and 95 °C for 2 min, which is followed by 40 cycles composed of denaturation at 95 °C for 15 s and annealing at 60 °C for 1 min. Standard curves for mFcRn and hFcRn were constructed from the isolated cloned mFcRn and hFcRn plasmid respectively. The relative standard curve for the control 18s was constructed from serial dilution of control mice liver cDNA. The standard curves were constructed for every tissue sample analysis. The cycle threshold (Ct) value was determine using MxPro QPCR software (Agilent Technologies) and the amount of mFcRn and hFcRn cDNA in tissue samples were calculated from a standard curve. The amount of mFcRn and hFcRn cDNA was expressed as a copy number per µg of extracted RNA normalized by a control 18s within each tissue.

### 2.7. Protein Extraction

The protein was extracted from various tissues (liver, lung, spleen, gastrointestinal (GI), kidney, heart, muscle, and skin) obtained from Swiss Webster and transgenic mice and from adult human liver and small intestine tissues. Approximately 20 mg of tissue was placed in the reservoir of a Dounce Tissue Grinder. Additionally, 500 μL of RIPA lysis buffer (Thermo Scientific, Rockford, IL, USA) with a protease inhibitor (Thermo Scientific) was added to the tissue grinder and the tissue was homogenized. Homogenate was transferred to micro centrifuge tubes and the reservoir of the tissue grinder was rinsed with an additional 500 µL of fresh lysis buffer. The rinsing solution was transferred to the micro centrifuge tubes, which brings the final volume of tissue homogenate to approximately 1 mL. Tissue homogenates were incubated at 4 °C for 1 h with agitation. Undissolved tissue debris was pelleted by centrifuging at 14,000 RCF for 20 min at 4 °C. The homogenate supernatant was aliquoted into new micro centrifuge tube and stored at −80 °C until used. The total protein concentration of the tissue extract was determined by using the Bradford assay (Sigma-Aldrich).

### 2.8. Preparation of Background Matrix Stock

The protein was extracted from transgenic mice tissue or C57BL/6J mice tissue samples using RIPA buffer. Approximately 20 mg of transgenic or C57BL/6J mouse tissue was weighed out and placed in the reservoir of the Dounce Tissue Grinder with 500 μL of RIPA lysis buffer containing protease inhibitors. The tissues were homogenized and the homogenates were transferred to micro centrifuge tubes. The reservoir of the tissue grinder was rinsed with an additional 500 μL of fresh RIPA lysis buffer and the rinsing solution was added to the micro centrifuge tubes. The tissue homogenates were incubated at 4 °C for 1 h with agitation. Undissolved tissue debris was pelleted by centrifuging at 14,000 RCF for 20 min at 4 °C. Tissue homogenate supernatants were aliquoted into new micro centrifuge tubes and stored at −80 °C until used.

### 2.9. Preparation of mFcRn Standards for the Assessment of FcRn Expression in Swiss Webster Mouse Tissues

Six standards at various concentrations were prepared from recombinant mFcRn (R&D Systems, Minneapolis, MN, USA). Reconstituted recombinant mFcRn at 250 ng/µL was diluted with sterile phosphate-buffered saline (PBS) to 250 ng/µL and stored at −80 °C until used. The same aliquot of recombinant mFcRn was used to prepare both standards and quality control (QC) stocks for each sodium dodecyl sulfate-polyacrylamide gel electrophoresis (SDS-PAGE) run. Two dilute working solutions (0.25 ng/µL and 0.05 ng/µL) were prepared freshly for each SDS-PAGE run from an aliquot of recombinant mFcRn using the transgenic mice background matrix stock. The transgenic mice background matrix was used because transgenic mice lacks the mFcRn protein. Standards were prepared from the two dilute working solutions, PBS, and reducing sample buffer (Thermo Scientific).

### 2.10. Preparation of hFcRn Standards for the Assessment of FcRn in Human or Transgenic Mouse Tissues

Six standards at various concentrations were prepared from recombinant hFcRn, which were obtained from Janssen R&D (Raritan, NJ, USA). Recombinant hFcRn stock solution at 5900 ng/uL was diluted with an appropriate C57BL/6J tissue background matrix to 118 ng/µL. An additional dilute working stock solution at 1.18 ng/µL was prepared by using the same C57BL/6J mice background matrix. The C57BL/6J mice background matrix was used because C57BL/6J mice lacks the hFcRn protein. The 118 ng/µL working solution was used to prepare QC stocks. Standards were prepared with the 1.18 ng/uL working solution, PBS, and reducing sample buffer (Thermo Scientific).

### 2.11. Preparation of Quality Control Stocks

Three QC stocks at various concentrations were prepared by spiking in either recombinant mFcRn or hFcRn into corresponding transgenic mouse or C57BL/6J mouse tissue samples prior to homogenization and protein extraction. For each QC stock, approximately 20 mg of corresponding transgenic mouse or C57BL/6J tissue was weighed and homogenized (after the addition of mFcRn or hFcRn), which is described above. QC samples were prepared from the prepared QC stocks at three different concentrations using PBS and reducing sample buffer (Thermo Scientific).

### 2.12. SDS-PAGE

SDS-PAGE gel was equilibrated in SDS-PAGE running buffer for 5 min prior to loading. Standard QC samples along with predetermined amounts of Swiss Webster tissue, transgenic mouse tissue, or human tissue extract (unknown sample) were loaded onto an SDS-PAGE gel. The initial voltage for SDS-PAGE was 50 V for 10 min followed by separation at 100 V for 50 min.

### 2.13. Western Blot

Western blot transfer buffer (Tris-Glycine buffer with 20% methanol) was prepared beforehand and cooled to 4 °C. The SDS-PAGE gels were equilibrated in Western blot transfer buffer for 5 min. The polyvinylidene difluoride (PVDF) membrane was briefly soaked in methanol and then equilibrated in transfer buffer for 5 min. Proteins in the SDS-PAGE gel was transferred to a PVDF membrane at 40 volt for 90 min. Ice packs were used to maintain the temperature of the buffer during the transfer process. After transfer, the membrane was blocked with 5% milk in TBST (Tris buffered saline with 0.1% tween 20) for 1 h at room temperature. After blocking, the membrane was probed with 2.5 µg/mL of goat anti-mFcRn antibody (R&D Systems) in TBST with 5% milk overnight at 4 °C or 6.8 µg/mL of 7E9 (mouse anti-hFcRn antibody) in TBST with 5% milk to probe for mFcRn or hFcRn, respectively. The membranes were also probed with rabbit anti-Vinculin antibody (Abcam, Cambridge, MA, USA) at 0.05 ug/mL, which serves as the loading control for each Western blot run. The membrane was washed four times with TBST and probed with 1 µg/mL of the horseradish peroxidase (HRP)-conjugated donkey anti-goat IgG antibody (Millipore, Billerica, MA, USA) in TBST with 5% milk or 1 µg/mL of HRP-conjugated goat anti-mouse IgG antibody (Millipore) in TBST with 5% milk. After 90 min of secondary antibody incubation, the membrane was washed 5 times with TBST and incubated in SuperSignal West Pico Chemiluminescent Substrate (Thermo Scientific) for 5 min. Lastly, the membrane was analyzed by using a ChemiDOC XRS system (Bio-Rad). The standard curve was constructed from densitometry analysis of standards. The accuracy and precision of the assay was evaluated through the use of the three QCs with FcRn concentrations determined from back-extrapolation based on the standard curve. FcRn protein concentrations in unknown samples were normalized within each blot with the use of the loading control (vinculin). Weight-normalize FcRn protein concentration in tissue is calculated by the equation below,

$$\;C_{\mathrm{FcRn},\mathrm{tissue}}=\frac{X_{\mathrm{FcRn}}}{X_{\mathrm{total}\;\mathrm{protein},\;\mathrm{Western}\;\mathrm{Blot}}}*\frac{C_{\mathrm{total}\;\mathrm{protein},\;\mathrm{tissue}\;\mathrm{extract}}{*V}_{\mathrm{tissue}\;\mathrm{extract}}}{V_{\mathrm{tissue}}}\;$$

where C_FcRn,tissue_ is tissue weight-normalize FcRn concentration. X_FcRn_ is the amount of FcRn measured through a Western blot. X_total protein,Western Blot_ is the amount of the total protein loaded onto a Western blot. C_total protein,tissue extract_ is the total protein concentration in tissue extract. V_tissue extract_ is the volume of tissue extract. The V_tissue_ is the volume of tissue used in protein extraction (calculated using the weight of tissue and assuming tissue density of 1 g/mL).

### 2.14. Western Method Validation

Validation of the quantitative Western blot method was done on a tissue by a tissue basis. First, the quantitative Western blot method was validated for muscle tissue using standards constructed from transgenic mice muscle tissue and QCs prepared with recombinant mFcRn protein. For the remaining tissues, an initial evaluation was made to determine whether accurate recovery could be achieved with the use of a standard curve generated with the use of muscle tissue. In this evaluation, a single Western blot analysis was performed for each tissue with standards constructed from transgenic mice muscle tissue and with QCs prepared from transgenic mice muscle tissue and with the tissue under evaluation. If QC recovery for each tissue was within 20% of nominal values, then further analyses of that tissue was based on standards prepared with transgenic mice muscle tissue. If the error for QCs prepared for the tissue under evaluation was >20%, then the subsequent validation work was performed by using standards prepared from the tissue under evaluation. The quantitative Western blot method was also validated for the quantification of hFcRn in human tissue and transgenic mice tissues by using C57BL/6J mice tissues for the background matrix.

### 2.15. Identification of Human FcRn in Transgenic Mouse Tissue Extract

Various human FcRn transgenic mouse tissue extracts (muscle, lung, kidney, heart, and GI) were separated on the SDS-PAGE gel and transferred onto the PVDF membrane as described earlier. The membrane was blocked with 5% milk in TBST for 1 h at room temperature. After blocking, the membrane was probed with 1 µg/mL of rabbit anti-hFcRn antibody (Abcam) or 6.8 µg/mL of 7E9 (mouse anti-hFcRn mAb) in TBST with 5% milk. The membrane was washed 4 times with TBST and probed with 0.2 µg/mL of HRP-conjugated goat anti-rabbit IgG antibody (Millipore) in TBST with 5% milk or 1 µg/mL of HRP-conjugated goat anti-mouse IgG antibody (Millipore) in TBST with 5% milk. After 90 min under a secondary antibody incubation, the membrane was washed 5 times with TBST and incubated in the SuperSignal West Pico Chemiluminescent Substrate (Thermo Scientific) for 5 min. Lastly, the membrane was analyzed with the use of a ChemiDOC XRS system.

### 2.16. Mathematical Analysis and Statistical Evaluation

Tissue abundance of FcRn mRNA was compared among different tissues of transgenic mice, Swiss Webster mice, and humans. Tissue FcRn abundance was presented as a copy number of FcRn mRNA per µg of extracted RNA. The relative abundance of FcRn mRNA was analyzed by comparing the mean copy number of FcRn mRNA.

Similarly, tissue abundance of the FcRn protein was compared among different tissues of transgenic mice, Swiss Webster mice, and humans. FcRn protein concentrations in tissue extracts determined through Western blot analysis were converted to tissue average concentrations by assuming a tissue extract volume of 1 mL and tissue density of 1 g/mL. As such, the tissue average FcRn concentration was calculated by using the equation below.

C_t,avg_ = C_FcRn,extract_ ∗ C_extract_ ∗ M_tissue_ ∗ density

where C_t,avg_ is the tissue average concentration in nM. C_FcRn,extract_ represents FcRn concentration in tissue extract expressed as moles of FcRn per µg of extracted protein and determined through Western blot analysis. C_extract_ represents the total protein concentration in the tissue extract and M_tissue_ represents the amount of tissue used to prepare each tissue extract. The densities of mouse and human tissues were assumed to be 1 g/mL. The relative abundance of the FcRn protein was analyzed by comparing the mean tissue average concentration.

The correlation of FcRn mRNA and protein expression in Swiss Webster mice, transgenic mice, and human tissue were assessed with regression analysis. FcRn mRNA and protein expression data from individual Swiss Webster mice, transgenic mice, and human subjects were used in the regression analysis. The regression analysis was performed using Minitab (Minitab Inc., State College, PA, USA).

### 2.17. Evaluation of FcRn Turnover in Human Umbilical Cells

The human umbilical vein cell line, EA.hy926, was purchased from ATCC and grown in DMEM (Invitrogen) supplemented with 10% fetal bovine serum (FBS) (Invitrogen) and 1% penicillin/streptomycin (Invitrogen) in a humidified atmosphere of 5% CO_2_ at 37 °C. Sixteen T-75 flasks containing confluent EA.hy926 cells were used in the labeling experiment (described below). Existing media was replaced with DMEM, no glutamine, no methionine, no cysteine (Invitrogen), supplemented with 63 mg/L l-cystine (Invitrogen), 584 mg/kg l-glutamine (Invitrogen), 10% FBS, and 1% penicillin/streptomycin, and incubated in an incubator for 15 min to deplete intracellular methionine. After the 15 min of incubation, each flask was washed twice with DMEM, no glutamine, no methionine, and no cysteine, supplemented with 63 mg/L l-cystine, 584 mg/L l-glutamine (Invitrogen), 10% FBS, and 1% penicillin/streptomycin along with EasyTag l-[35S]-methionine to achieve radioactivity of 45 µCi/mL. After the two washed cells were incubated in the same wash medium for 90 min to allow the incorporation of [35S]-methionine into the cellular protein. After the 90 min of incubation, each T-flask was washed twice with DEME, HEPES (Invitrogen), supplemented with 750 mg/L methionine (Invitrogen), 63 mg/L l-cystine, 584 mg/kg l-glutamine, 10% FBS, and 1% penicillin/streptomycin, and incubated with the same wash medium in the closed T-flask system. Cells from 3 T-flasks were harvested at 0, 1, 3, 8, 12, and 24 h. Harvested cells were lysed using RIPA lysis buffer with a protease inhibitor. Cell lysate was stored at 4 °C until use.

FcRn was immuno-precipitated from cell lysate using Pierce direct magnetic IP and Co-IP kit (Thermo Scientific). 7E9 (a mouse anti-hFcRn antibody) was conjugated to the magnetic beads provided by the kit. The cell lysate was added to the 7E9 conjugated beads and incubated on an orbital shaker at room temperature for 2 h. After the 2 h incubation, the magnetic beads were separated from cell lysate. The magnetic beads were then washed twice with lysis buffer provided by the kit and once with ultrapure water. FcRn was eluded from the 7E9 conjugated beads using elusion buffer provided by the kit.

SDS-PAGE gels were equilibrated in SDS-PAGE running buffer for 5 min prior to loading. FcRn samples immuno-precipitated from cell lysates were loaded onto SDS-PAGE gels. The initial voltage for SDS-PAGE was 50 V for 10 min, which was followed by separation at 100 V for 50 min. After SDS-PAGE separation, the gels were dried using the DryEase Mini-Gel Drying System (Life Technologies, Grand Island, NY, USA). The gels were equilibrated in Gel-Dry Drying Solution provided by the kit. The gels were then sandwiched between two sheets of cellophane and mounted between the DryEase Gel Drying Frame. The mounted gels were allowed to air-dry for 24 h. Once the gels were dried, the gels were removed from the frame and wrapped in clear plastic wrap.

Dried gels were placed on a BAS storage phosphor screen (BAS Storage Phosphor Screen, Pittsburg, PA, USA) and exposed for 21 h. After the 21 h exposure, the signal captured on the BAS storage phosphor screen was detected with a FujiFilm BAS-5000 instrument. Densitometry analysis was performed using ImageJ software (available for download from National Institutes of Health, Bethesda, MD, USA).

Signals acquired from densitometry analysis at each time point were normalized to the average of signals from 0 h samples and expressed as percent 35S-methionine labeled FcRn remaining. Non-compartmental analysis (NCA) was performed on the transformed data and then the bi-exponential model was fitted to the data using the Phoenix WinNonlin (Certara, Princeton, NJ, USA).

## 3. Results

### 3.1. Quantification of Tissue FcRn mRNA Expression

**Figure 1:**
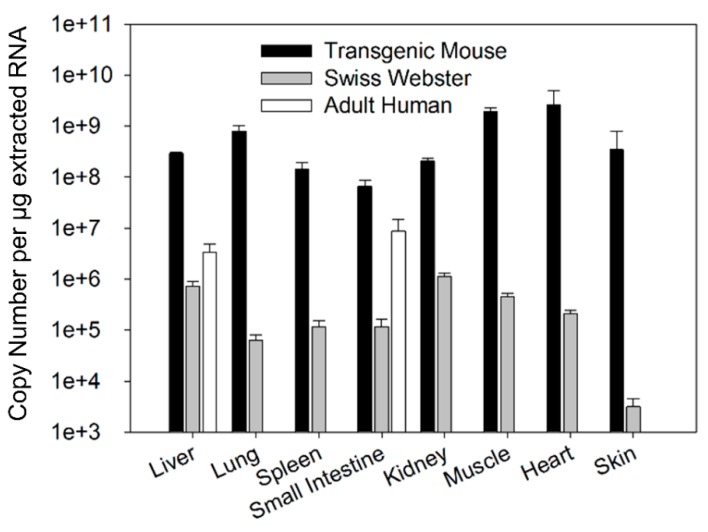
The comparison of FcRn mRNA expression among Swiss Webster mice, transgenic mice, and select human tissues. Data are expressed in terms of copy numbers per μg of extracted RNA. Data are mean ± standard deviation (SD) from 5 Swiss Webster mice, 3 transgenic mice, liver samples from 3 adult human subjects, and small intestine samples from 3 adult human subjects.

FcRn mRNA expression in tissues of Swiss Webster, transgenic mice, and humans were compared (Figure 1). The expression of human FcRn mRNA in transgenic mice was much higher when compared to the expression of mouse FcRn mRNA in Swiss Webster mice. The greatest difference in expression was seen in skin where the expression of human FcRn mRNA in transgenic mice was 109,000-fold higher when compared to mouse FcRn mRNA expression in Swiss Webster mice (Table 1). The smallest difference in expression was seen in the kidney where expression of human FcRn mRNA in transgenic mice was 184-fold higher when compared to mouse FcRn mRNA expression in Swiss Webster mice (Table 1). In addition to the significant difference in FcRn mRNA expression, the tissue selectivity of the FcRn expression differed substantially for human FcRn mRNA expression in transgenic mice vs. mouse FcRn mRNA in Swiss Webster mice. In transgenic mice, human FcRn expression was the lowest in the small intestine and the highest in the heart with rank ordering of mRNA expression as follows: small intestine < spleen < kidney < liver < skin < lung < muscle < heart. In contrast, in Swiss Webster mice, mouse FcRn expression was the lowest in skin and the highest in the kidney with rank ordering of mRNA expression as follows: skin < lung < small intestine < spleen < muscle < heart < liver < kidney. In adult human tissue, FcRn mRNA expression seen in the liver and the small intestine fall in between FcRn mRNA expression observed in Swiss Webster and transgenic mice.

### 3.2. Quantification of Tissue FcRn Protein Expression

**Figure 2:**
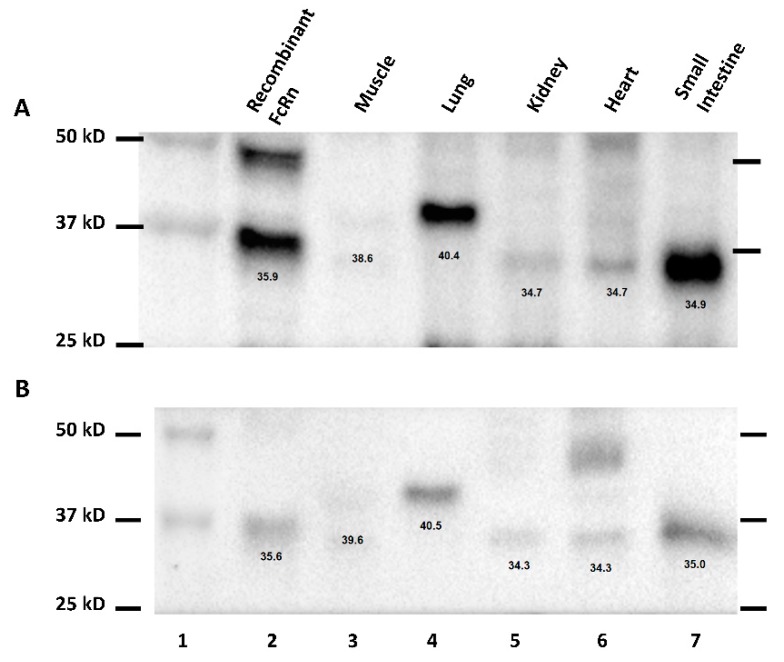
Identification of the human neonatal Fc receptor (hFcRn) band in transgenic mouse tissues. Various hFcRn transgenic mouse tissues probed with anti-hFcRn antibody (**A**) or 7E9 (**B**). Lane 1 is a molecular weight marker. Lane 2 is a recombinant hFcRn spiked into wild type mouse liver tissue extract. Lane 3–7 are human FcRn transgenic mouse muscle, lung, kidney, heart, and gastrointestinal (GI) tissue extract. Calculated molecular weight of each band is displayed below the corresponding band.

Probing of human FcRn transgenic mouse tissue extract for human FcRn using 7E9 yielded multiple bands at various molecular weight. To identify the band representing human FcRn, human FcRn transgenic mouse tissue extract was probed with a commercially available anti-hFcRn antibody in addition to 7E9 (Figure 2). Side-by-side comparison of Western blot images probed with two different anti-hFcRn antibody shows that recombinant hFcRn protein resolved at approximately 36 kD. Human FcRn in some tissue (kidney, heart, and GI) resolved at approximately 35 kD. In other tissues (muscle and lung), human FcRn resolved at approximately 40 kD. Additional bands that are not present in both Western blots probed with two different anti-hFcRn antibodies represent non-specific or unintended interaction of primary or secondary antibodies to components of tissue extract. As such, the 35 kD and 40 kD bands were identified to be human FcRn. 

**Figure 3:**
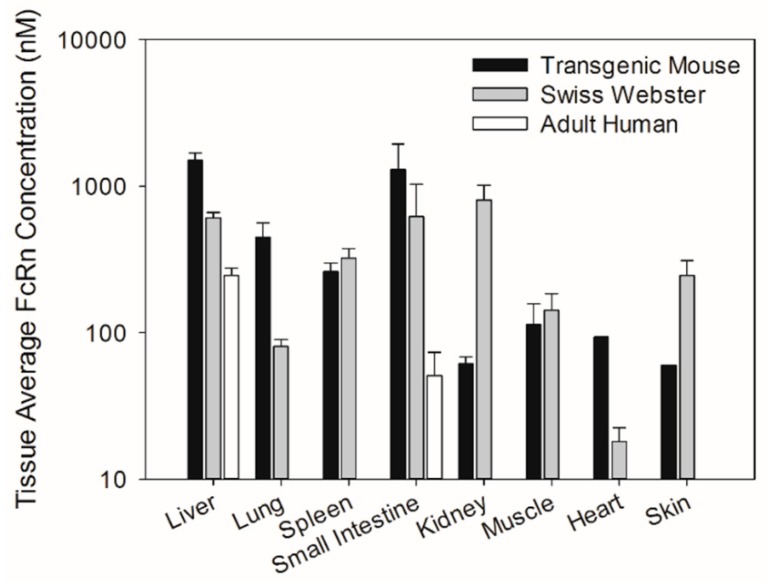
Comparison of tissue FcRn protein expression among Swiss Webster mice, transgenic mice, and humans. The data are expressed in tissue average FcRn concentration. Data are mean ± SD from 5 Swiss Webster mice, 3 transgenic mice, liver samples from 3 adult human subjects, and GI samples from 3 adult human subjects.

For the quantification of mouse FcRn in Swiss Webster mouse muscle, lung, spleen, and heart, the use of standards prepared from muscle tissue allowed for accurate recovery and quantification of mouse FcRn. However, quantification of mouse FcRn in the liver, small intestine, kidney, and skin required use of standards prepared from these tissues. For quantification of human FcRn in transgenic mouse muscle and lung, the use of standards prepared from muscle tissue allowed for accurate recovery and quantification of human FcRn. However, quantification of human FcRn in the liver, spleen, heart, small intestine, kidney, and skin required the use of standards prepared from these tissues. Muscle tissue was selected as the background matrix for the quantification of the stated tissues because of the availability of large quantities of muscle and due to the finding that this matrix allowed precise and accurate recovery of recombinant FcRn (spiked) in quality control samples during the method validation (Supplementary Materials). Due to the limitation of the Western blot assay and low protein extraction efficiency of skin tissue, human FcRn abundance in the skin tissue of transgenic mice cannot be determined. Based on the limit of quantification of the Western blot assay, skin tissue FcRn concentration is estimated to be below 60 nM. The FcRn protein expression in tissues of Swiss Webster, transgenic mice, and human were compared (Figure 3). There were smaller differences in FcRn protein expression between Swiss Webster and transgenic mice relative to the differences observed in mRNA expression. Human FcRn protein expression in transgenic mice was found to be 5.6-fold higher in lung tissue to 13-fold lower in kidney tissue than mouse FcRn protein expression in Swiss Webster mice (Table 2). Similar to the FcRn mRNA tissue expression pattern, the tissue selectivity of the FcRn protein expression differed between transgenic and Swiss Webster mice. In transgenic mice, human FcRn protein expression was the lowest in the skin and the highest in the liver with rank ordering of protein expression as follows: skin < kidney < muscle < heart < spleen < lung < small intestine < liver. In contrast, in Swiss Webster mice, mouse FcRn protein expression was the lowest in muscle and the highest in kidney tissues with rank ordering of protein expression as follows: muscle < lung < heart < skin < spleen < small intestine < liver < kidney. In adult humans, the liver showed significantly higher FcRn protein expression compared to human small intestine tissue (Table 3). FcRn protein expression in the human liver and the small intestine were significantly lower compared to FcRn protein expression in Swiss Webster and transgenic mice. Lastly, no significant differences in FcRn protein expression were found in human liver and small intestine tissues when compared across different age groups (Table 3).

**Figure 4:**
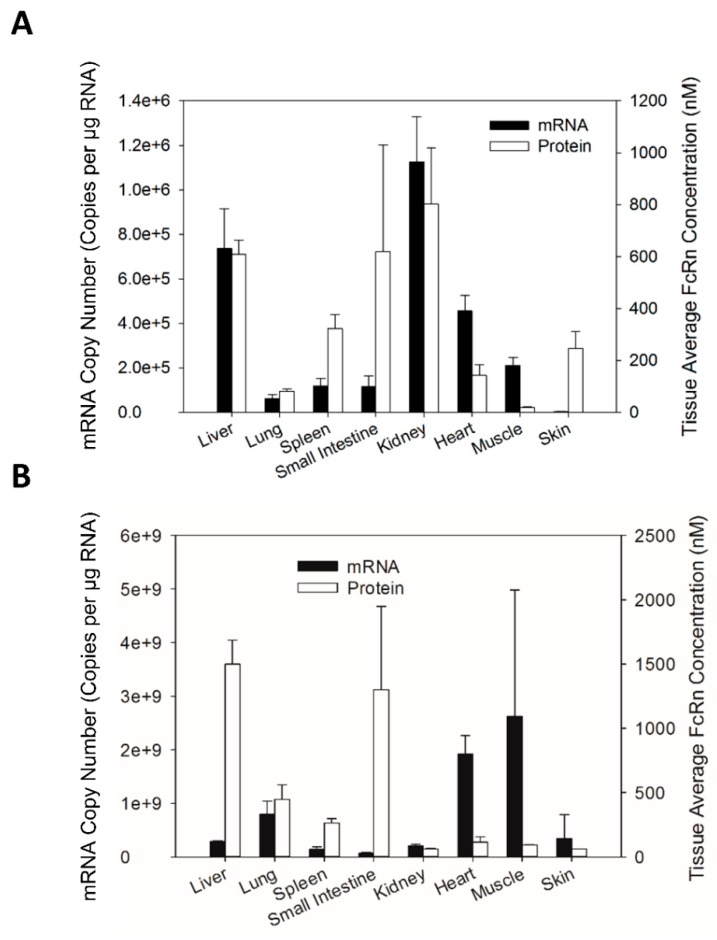
Comparison of tissue mFcRn mRNA and protein expression pattern in Swiss Webster mice (**A**) and comparison of tissue hFcRn mRNA and protein expression pattern in transgenic mice. (**B**) Data are mean ± SD from 5 Swiss Webster mice and 3 transgenic mice.

**Figure 5:**
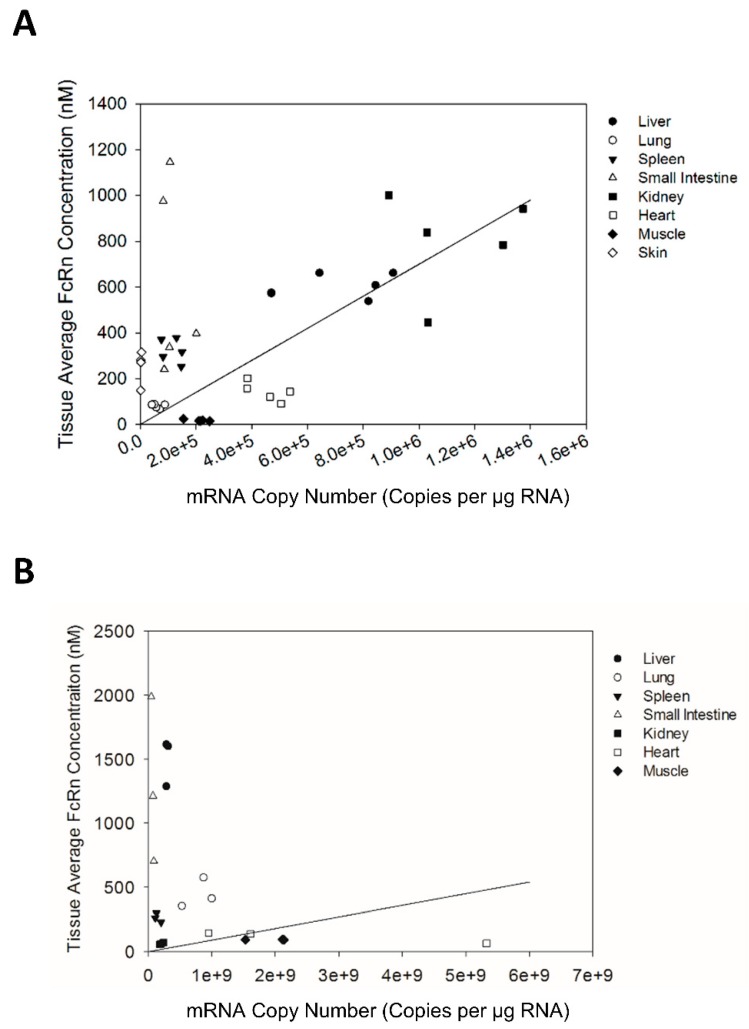
Correlation between mFcRn mRNA and protein expression in Swiss Webster mice (**A**) and transgenic mice (**B**). Individual mouse tissue mRNA abundance plotted against tissue protein abundance. Regression analysis showed significant (*p* < 0.0005) correlation between tissue FcRn mRNA and protein expression in Swiss Webster mice with *R*^2^ value of 0.1131. Regression analysis showed no significant correlation between tissue FcRn mRNA and protein expression in transgenic mice.

The tissue expression patterns of FcRn mRNA and protein were compared for Swiss Webster mice as well as transgenic mice (Figure 4). In general, the tissue FcRn protein expression pattern was similar but not very highly correlated with the tissue FcRn mRNA expression pattern for Swiss Webster mice. To further investigate the correlation of FcRn mRNA and protein expression, FcRn protein abundance in each tissue was plotted against FcRn mRNA abundance in each tissue for every individual mouse. Regression analysis indicated that FcRn protein abundance significantly correlated with FcRn mRNA abundance for Swiss Webster but not for transgenic mice (Figure 5).

### 3.3. Quantification of FcRn Turnover Kinetics

**Figure 6:**
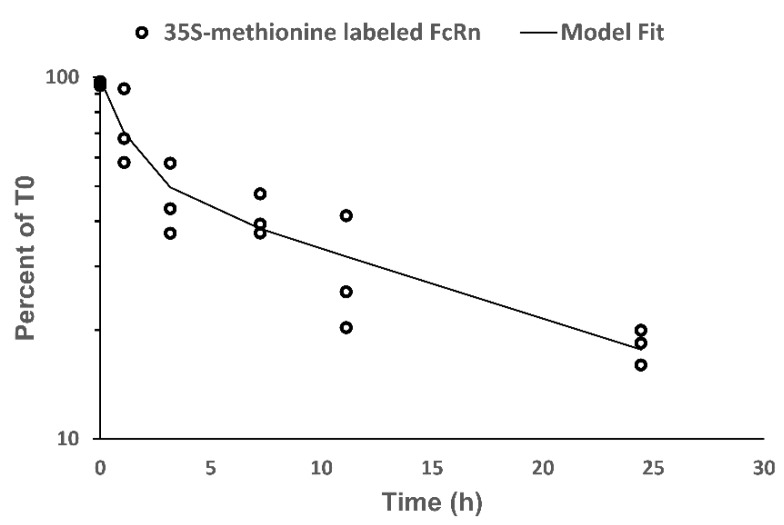
Degradation of 35S-methionine labeled FcRn in EA.hy926 cells. Decline in 35S-methionine labeled FcRn with time is expressed as a percent of labeled FcRn at 0 h. Each data point represents cells collected from a single T-flask. The fitting of a bi-exponential model to the data is shown by a solid line.

In EA.hy926 cells, 35S-methionine labeled FcRn demonstrated a biphasic decline (Figure 6). The estimated mean residence time from NCA analysis was 21.9 h, which translates to an apparent half-life of 15.2 h. The bi-exponential model fitted the 35S-methionine labeled FcRn degradation data well. The fitted model parameters are shown in Table 4. The biphasic decline in 35S-methionine FcRn corresponds to a shorter half-life of 0.912 h and a longer half-life of 15.6 h, which suggests that FcRn may exist in two kinetically-distinct pools.

## 4. Discussion

Human FcRn transgenic mouse models have been developed to provide more suitable preclinical rodent models for the evaluation of therapeutic mAbs. However, how accurately the expression pattern of human FcRn in the transgenic mice mimics that of wild type mice and humans has not been assessed to date. Furthermore, the human FcRn transgenic mouse model expresses human FcRn α-chain and mouse FcRn β_2_-microglobulin. Praetor and Hunziker have demonstrated the absence of the beta-2-microglobulin decreases among IgG binding to FcRn compared to native FcRn, which suggests beta-2-microglubulin is involved in IgG binding [17]. Therefore, the chimeric FcRn receptor expressed by the transgenic mouse model may have different affinity for human IgG compared to the fully human FcRn receptor. The present investigation focuses on assessing differences in tissue FcRn mRNA and protein expression between a human FcRn transgenic mouse model, Swiss Webster mice, and select human tissues. Swiss Webster mice were chosen because this strain is commonly employed in preclinical assessments of mAb pharmacokinetics. Based on our qPCR and Western blot analyses, the expression pattern of human FcRn in transgenic mice differs significantly from that of Swiss Webster mice and both Swiss Webster and transgenic mice tissue FcRn expression were significantly higher than FcRn expression in human liver tissue and small intestine tissue.

During the development of the Western method for the quantification of hFcRn in transgenic mouse tissue, multiple bands were identified. Additionally, hFcRn in muscle and lung tissue lysates of transgenic mice were detected at a slightly higher molecular weight when compared to other tissues. The difference in molecular weight of FcRn detected in different tissues may be the result of different glycosylated forms of FcRn. Haymann et al. have detected multiple bands corresponding to hFcRn in the human kidney, which may potentially relate to several glycosylated forms of FcRn [18]. Further investigations are needed to assess the existence of tissue or cell type-specific differences in FcRn glycosylation.

The relevance of these differences is uncertain at present. However, it is clear that FcRn expression is a major determinant of mAb plasma and tissue pharmacokinetics [19,20,21]. FcRn expression differences between rodent models and humans may, in theory, significantly impact the utility of the models in predicting key pharmacokinetic attributes (e.g., subcutaneous bioavailability) and, potentially, impact assessments of tissue selectivity, efficacy, and toxicity. For example, data collected in FcRn knockout mice suggests that FcRn plays a key role in the subcutaneous (s.c.) bioavailability of mAb [22,23]. Additionally, Deng et al. found that FcRn binding affinity correlated directly with the s.c. bioavailability of a series of mAb in mice where mAb with low FcRn affinity showed low bioavailability while mAb with high affinity for FcRn exhibited high bioavailability [24]. Given the finding that transgenic mice express significantly lower FcRn concentrations in skin tissue relative to wild type mice, transgenic mice may be expected to show lower s.c. bioavailability for mAb than wild type mice. Additionally, FcRn is functionally expressed on the epithelial cells in lung and intestine tissue [25,26,27]. FcRn expressed on mucosal surfaces in these tissues functions to transport IgG across mucosal epithelial membranes [25,26,27]. There has been interest in the development of mAbs and Fc-fusion proteins for pulmonary delivery that capitalizes on the FcRn-mediated transport [28]. The higher FcRn expression in the lungs of transgenic mice may impact the absorption and bioavailability of mAbs and Fc-fusion proteins.

Differences in the expression pattern of FcRn between models and humans may impact the tissue selectivity of mAb disposition. Investigations of the tissue distribution of IgG in wild-type and FcRn knockout mice has demonstrated that fat, skin, and muscle tissue showed a decrease in tissue-to-blood exposure ratios for IgG while the liver, spleen, and lung showed an increase in a tissue-to-blood exposure ratio of IgG in FcRn knockout mice compared to wild type mice [19]. Significant differences in tissue FcRn expression between transgenic mice, Swiss Webster mice, and humans may lead to inter-species differences in the disposition pattern of mAb and contribute to uncertainties when making human mAb PK/PD predictions based on data collected in animal models.

There has been a growing interest in understanding variability in FcRn expression and its contribution to variability in antibody disposition in humans. If FcRn mRNA expression can be shown to be a good predictor for FcRn protein expression, then assessments of inter-individual variability in FcRn expression may be simplified (due to the relative ease of assessments of mRNA expression by qPCR vs. assessments of protein expression). Comparison of the tissue FcRn mRNA and protein expression pattern in Swiss Webster mice indicates that the tissue FcRn mRNA expression pattern correlates significantly with the expression of the FcRn protein. However, although the correlation between FcRn mRNA and protein expression is statistically significant, only 11% of the variability in FcRn protein expression is explained by the linear model based on the *R*^2^ value of 0.113. As such, it may not be possible to employ FcRn mRNA expression to predict quantitatively FcRn protein expression in human tissue samples. 

Anti-FcRn mAb are under development by several pharmaceutical companies for possible use in the treatment of auto-immune and allo-immune conditions. The expression and turnover of FcRn may be expected to be a determinant of the dose-potency and efficacy of anti-FcRn therapy. Published evaluation of anti-FcRn mAb in rats and monkeys demonstrates much greater dose potency in terms of accelerating IgG elimination and decreasing endogenous IgG concentrations than shown in hFcRn transgenic mouse models [14,29,30]. The high tissue FcRn expression found in the Tg276 strain of human FcRn transgenic mouse may explain, in part, prior observations of poor dose potency of anti-hFcRn mAb in this strain [30]. The very high expression of human FcRn in transgenic mouse models likely decreases the utility of these models in evaluating anti-hFcRn pharmacokinetics, pharmacodynamics, efficacy, and dose-potency. 

A recently published study by Fan et al. investigated tissue FcRn expression in the Tg32 strain of human FcRn transgenic mice [11]. The Tg276 strain used in the present investigation expresses human FcRn α-chain under the control of the human cytomegalovirus and the immediate early promoter/enhancer with the chicken beta-actin/rabbit beta-globin hybrid promoter (CAG). Unlike the Tg276 strain, the entire human FcRn gene is knocked in under the control of the natural human promoter in the Tg32 strain. The CAG promoter used in the Tg276 strain is a strong synthetic promoter frequently used to drive high levels of gene expression. Therefore, it is expected that the Tg276 strain would have much higher mRNA expression compared to the Tg32 strain of transgenic mice or Swiss Webster mice. The higher mRNA expression in the Tg276 strain also translates to higher protein expression. Fan et al. reported that the tissue average FcRn concentration across the analyzed tissues in homozygous Tg32 transgenic mice ranges from 7.96 nM to 111.2 nM. In heterozygous Tg32 transgenic mice, tissue FcRn concentration ranges from 3.51 nM to 55.38 nM [11]. The tissue average FcRn concentrations presented by Fan et al. is generally lower than the tissue average FcRn concentration measured in corresponding tissues of Swiss Webster mice presented in the current study. For muscle and lung tissues, the tissue average FcRn concentration in homozygous Tg32 transgenic mice reported by Fan et al. (15.67 nM and 98.30 nM for muscle and lung) are very similar to values observed in Swiss Webster mice (18.0 nM and 88.3 nM for muscle and lung). In other tissues, the tissue average FcRn concentration in homozygous Tg32 transgenic mice reported by Fan et al. are significantly lower than values observed in Swiss Webster mice. Both homozygous and heterozygous Tg32 strains of human FcRn transgenic mice have significantly lower tissue average FcRn concentration compared to the Tg276 strain of transgenic mice. Additionally, FcRn expression in the liver of homozygous Tg32 mice was significantly lower than FcRn expression in human liver tissue (77.64 nM vs. 247 nM). In the small intestine, FcRn expression was quite similar between Tg32 mice and humans (56.77 nM vs. 51 nM). Of note, Fan et al. perfused mouse tissues prior to quantification of FcRn expression while tissues (human and mouse) were not perfused within the present work. Perfusion may be expected to lead to reductions in the measured amount of FcRn mRNA or protein in tissues by wholly or partially removing FcRn found within hematopoietic cells residing within vascular spaces. However, perfusion is not expected to remove hematopoietic cells from tissue stroma beyond the blood vessels and the quantitative significance of perfusion on measured FcRn concentrations has not yet been investigated.

In another recently published study, Latvala et al. qualitatively compared FcRn expression in various cell types from different tissues (e.g., hepatocytes, goblet cells, neuronal cells, epithelial cells, keratinocytes, cardiomyocytes, myocytes, macrophages, and T lymphocytes) across several preclinical species (monkey, rat, wild-type mouse, SCID mouse, Tg32, and Tg276 strain of human FcRn transgenic mouse) and human [12]. Monkeys, rats, and humans have similar relative FcRn expression across cell types and tissues. The wild type and the SCID T32 strain of transgenic mice have lower relative FcRn expression in several cell types and tissues compared to humans. The T276 strain of transgenic mice have higher relative FcRn expression while the Tg32 strain of transgenic mice have lower relative FcRn expression compared to humans. Based on the published investigation into tissue FcRn expression by Fan et al., Latvala et al. and our quantitative assessment of both the Tg32 strain and the Tg276 strain of human FcRn transgenic mice may not provide a good representation of FcRn expression in human tissues. Rats and monkeys likely provide the closest representation of FcRn expression in human tissue. The present investigation did not investigate FcRn expression in different cell types or the contribution of different cell types to the measured FcRn expression in tissues. FcRn expression in cells other than vascular endothelial cells and hematopoietic cells has been reported [27,31,32] and Latvala et al. have demonstrated that different cell types in the same tissue or organ can have different FcRn expression [12]. The heterogeneous expression of FcRn by different cell types in the same tissue may explain, in part, the poor correlation between FcRn mRNA and protein expression. For example, it is possible that the relatively small tissue samples used in the present work fail to provide an accurate representation of the combined population of cell types in tissue and it is possible that different distributions of cells were associated with samples that were analyzed for mRNA and for protein analyses. Nonetheless, the finding of a relatively poor correlation between FcRn mRNA and protein in tissue samples and the highly variable mRNA concentrations measured in tissues indicates that use of small tissue samples (e.g., collected via biopsy) and assessment of mRNA expression may fail to provide a quantitatively useful surrogate for FcRn protein expression. 

The human FcRn α-chain expressed in transgenic mouse models does not bind endogenous mouse IgG and, as a result, both the Tg276 and Tg32 transgenic mouse models have extremely low plasma IgG concentrations. Unlike the transgenic mouse models, plasma concentrations of endogenous IgG in humans are significantly higher. Given the higher plasma IgG concentration and species differences in IgG-FcRn binding, it is expected that FcRn receptors within cellular endosomes are saturated to a much higher extent in humans relative to the condition found in the transgenic mouse models. As such, although the Tg276 and Tg32 mouse models express human FcRn, the pharmacokinetics of mAb in the transgenic mouse models are not expected to allow direct, quantitative prediction of the efficiency of FcRn-mediated protection of mAb in human subjects. Additionally, FcRn expression differences between animal models and humans may be particularly important for the preclinical assessment of the pharmacokinetics, pharmacodynamics, and efficacy of anti-FcRn therapies (i.e., for treatment of autoimmunity). Given the high expression of hFcRn Tg276 mice, use of these mice may lead to substantial under-predictions of the utility and dose-potency of anti-FcRn therapies.

The present investigation demonstrated biphasic turnover kinetic of FcRn in EA.hy926 cells. We hypothesize that this finding relates to the intracellular distribution of FcRn within two kinetically-distinct pools. FcRn is known to be distributed throughout the endosomal system including within endosomes and Golgi [33,34]. Additionally, a small fraction of FcRn is associated with the plasma membrane [33]. D’Hooghe et al. have shown that a fraction of the cell surface FcRn is rapidly internalized while the remaining fraction shows significantly longer persistence [33]. Different trafficking dynamics of FcRn located on plasma membrane, in endosomes, and in Golgi may lead to rates of turnover of these FcRn pools, which explains the observed biphasic elimination of FcRn in EA.hy926 cells.

In conclusion, our study demonstrated that tissues of the B6.Cg-*Fcgrt*tm1Dcr Tg/Tg(*FCGRT*)276Dcr homozygous mouse model exhibited significantly different FcRn mRNA and protein expression when compared to the expression of FcRn in tissues obtained from Swiss Webster mice and humans. The implication of these differences on mAb PK/PD including the PK/PD of anti-FcRn mAb requires further investigation.

## Figures and Tables

**Table 1:** Expression of mouse neonatal Fc receptor (mFcRn) and human neonatal Fc receptor (hFcRn) mRNA in various tissues of Swiss Webster and transgenic mice.

	Copy Number (Mean (%CV))		Comparison (Fold Difference)
	Swiss Webster	Transgenic	Transgenic vs. Swiss Webster
Liver	7.37E5 (24.1)	2.90E8 (5.35)	394
Lung	6.26E4 (29.2)	7.97E8 (30.4)	12,723
Spleen	1.17E5 (30.3)	1.42E8 (34.2)	1213
Gastrointestinal	1.17E5 (41.6)	6.58E7 (32.8)	565
Kidney	1.13E6 (18.0)	2.07E8 (12.3)	184
Heart	4.56E5 (15.4)	1.92E9 (17.9)	4221
Muscle	2.12E5 (16.2)	2.62E9 (89.9)	12,404
Skin	3.14E3 (45.1)	3.42E8 (130.6)	108,889

Data are the copy number of mFcRn) and hFcRn mRNA per µg of extracted RNA (mean and percent coefficient of variation (%CV) in Swiss Webster and transgenic mice, respectively. Fold difference in mRNA expression represents the ratio of the mRNA copy number in transgenic mice to Swiss Webster mice.

**Table 2:** Expression of mFcRn and hFcRn protein in various tissues of Swiss Webster and transgenic mice.

	Tissue Average Concentration in nM (Mean (%CV))		Comparison (Fold Difference)
	Swiss Webster	Transgenic	Swiss Webster vs. Transgenic
Liver	609 (8.93)	1501 (12.3)	2.47
Lung	80.3 (11.6)	447 (25.6)	5.56
Spleen	323 (16.4)	263 (13.7)	0.81
GI	619 (66.4)	1300 (49.8)	2.10
Kidney	802 (27.0)	61.5 (10.8)	0.08
Heart	143 (29.5)	114 (38.6)	0.80
Muscle	18.0 (24.0)	92.9 (1.38)	5.16
Skin	246 (26.6)	60 ^a^	0.24 ^a^

The data are tissue average FcRn concentration in nM (mean and %CV) in Swiss Webster and transgenic mice, respectively. The fold difference in protein expression represents the ratio of protein concentration in transgenic mice to Swiss Webster mice. ^a^ Due to a limitation of the assay, FcRn concentration in skin tissue cannot be determined. Based on the assay’s limit of quantification, FcRn concentration in skin tissue is estimated to be less than 60 nM.

**Table 3:** Expression of FcRn protein in human liver and intestine tissue.

	>18 Years Old	3–12 Years Old	0–2 Years Old
Liver	247 (12.0)	437 (53.5)	337 (21.5)
Intestine	44 (62.2)		38 (28.5)

The data are from 3 human liver and 3 intestine tissue samples for each age group.

**Table 4:** Fitted model parameters.

Parameter	Mean	Standard Error
A (%)	46.4	11.3
B (%)	52.3	6.25
α (h^1^)	0.76	0.326
β (h^−1^)	0.0444	0.0075

## Supplementary Materials

The following are available online at http://www.mdpi.com/2218-273X/8/4/115/s1; Table S1: Yield and purity of RNA extracted from tissues of Swiss Webster mice; Table S2: Yield and purity of RNA extracted from tissues of transgenic mice; Table S3: Yield and purity of RNA extracted from adult human tissues; Figure S1: Integrity of RNA extracted from mouse and human tissues; Table S4: 28S:18S ratio of RNA samples extracted from representative mouse and human tissues; Figure S2: Standard curve for mouse FcRn RNA; Figure S3: Standard curve for human FcRn RNA; Figure S4: Representative validation of Western blot for mFcRn in Swiss Webster muscle tissue; Figure S5: Representative validation of Western blot for mFcRn in Swiss Webster lung tissue; Figure S6: Representative validation of Western blot for mFcRn in Swiss Webster spleen tissue; Figure S7: Representative validation of Western blot for mFcRn in Swiss Webster heart tissue; Figure S8: Representative validation of Western blot for mFcRn in Swiss Webster kidney tissue; Figure S9: Representative validation of Western blot for mFcRn in Swiss Webster small intestine tissue; Figure S10: Representative validation of Western blot for mFcRn in Swiss Webster liver tissue; Figure S11: Representative validation of Western blot for mFcRn in Swiss Webster skin tissue; Figure S12: Representative validation of Western blot for hFcRn in transgenic mouse muscle tissue; Figure S13: Representative validation of Western blot for hFcRn in transgenic mouse lung tissue; Figure S14: Representative validation of Western blot for hFcRn in transgenic mouse spleen tissue; Figure S15: Representative validation of Western blot for hFcRn in transgenic mouse heart tissue; Figure S16: Representative validation of Western blot for hFcRn in transgenic mouse kidney tissue; Figure S17: Representative validation of Western blot for hFcRn in transgenic mouse small intestine tissue; Figure S18: Representative validation of Western blot for hFcRn in transgenic mouse liver tissue; Figure S19: Representative validation of Western blot for hFcRn in transgenic mouse skin tissue.
