# Supplementary material for: FcRn Expression in Wildtype Mice, Transgenic Mice, and in Human Tissues
Source: Biomolecules. 2018 Oct 15;8(4):115. doi: 10.3390/biom8040115 (PMC6316262; doi:10.3390/biom8040115)
Supplement: Supplementary file 1 [file biomolecules-08-00115-s001.pdf]

Supplementary Materials:

Table S1. Yield and purity of RNA extracted from tissues of Swiss Webster mice

	Mouse ID	Yield (ng/µL)	Purity (A_{260}/A_{280})	Purity (A_{260}/A_{230})
**Liver**	M1	709	2.10	2.35
	M2	462	2.06	2.13
	M3	545	2.11	2.34
	M4	425	2.08	2.29
	M5	603	2.12	1.94
**Lung**	M1	158	2.12	2.15
	M2	384	2.12	1.70
	M3	248	2.11	2.27
	M4	185	2.11	2.15
	M5	168	2.12	1.98
**Spleen**	M1	614	2.15	2.25
	M2	443	2.09	2.26
	M3	580	2.14	2.22
	M4	599	2.14	2.26
	M5	284	2.08	1.49
**GI**	M1	463	2.09	2.13
	M2	460	2.08	2.03
	M3	374	2.09	2.06
	M4	674	2.14	2.29
	M5	337	2.09	1.85
**Kidney**	M1	287	2.13	1.77
	M2	307	2.13	2.24
	M3	405	2.11	1.85

	M4	306	2.11	2.26
	M5	544	2.15	2.28
**Muscle**	M1	221	2.08	2.21
	M2	146	2.07	1.67
	M3	212	2.06	2.23
	M4	201	2.09	2.19
	M5	176	2.08	2.19
**Heart**	M1	327	2.10	1.90
	M2	334	2.06	2.19
	M3	312	2.08	2.14
	M4	361	2.09	2.17
	M5	314	2.08	2.19
**Skin**	M1	219	2.01	1.76
	M2	65	2.04	1.83
	M3	95	2.10	2.12
	M4	109	2.10	2.22
	M5	111	2.05	2.22

Data are yield and purity of RNA extracted RNA from tissues of 5 Swiss Webster mice.

Table S2. Yield and Purity of RNA extracted from tissues of transgenic

	Mouse ID	Yield (ng/μL)	Purity (A_{260}/A_{280})	Purity (A_{260}/A_{230})
**Liver**	M1	427	2.04	2.10
	M2	665	2.13	2.15
	M3	723	2.15	1.99
**Lung**	M1	388	2.07	2.20
	M2	356	2.05	2.28
	M3	218	2.08	2.12
**Spleen**	M1	909	2.09	2.24
	M2	464	2.03	1.98
	M3	596	2.06	2.19
**GI**	M1	405	2.07	2.16
	M2	585	2.19	2.00
	M3	630	2.17	2.03
**Kidney**	M1	167	2.10	2.12
	M2	200	2.10	1.58
	M3	218	2.10	2.19
**Muscle**	M1	187	2.10	2.14
	M2	168	2.10	2.07
	M3	97	2.07	1.40
**Heart**	M1	413	2.06	2.20
	M2	307	2.11	2.25
	M3	337	2.08	2.25
**Skin**	M1	129	2.05	2.21
	M2	88	2.19	2.20
	M3	48	2.00	2.16

Data are yield and purity of RNA extracted RNA from tissues of 3 transgenic mice.

Table S3. Yield and purity of RNA extracted from adult human tissues

	Subject ID	Yield (ng/μL)	Purity (A_{260}/A_{280})	Purity (A_{260}/A_{230})
**Liver**	1	793	2.06	2.26
	2	367	2.1	2.16
	3	611	2.07	2.21
**GI**	4	335	2.12	1.58
	5	206	2.13	2.02
	6	454	2.06	1.88

Data are yield and purity of RNA extracted RNA from liver and small intestine tissues of 6 human subject.

y.

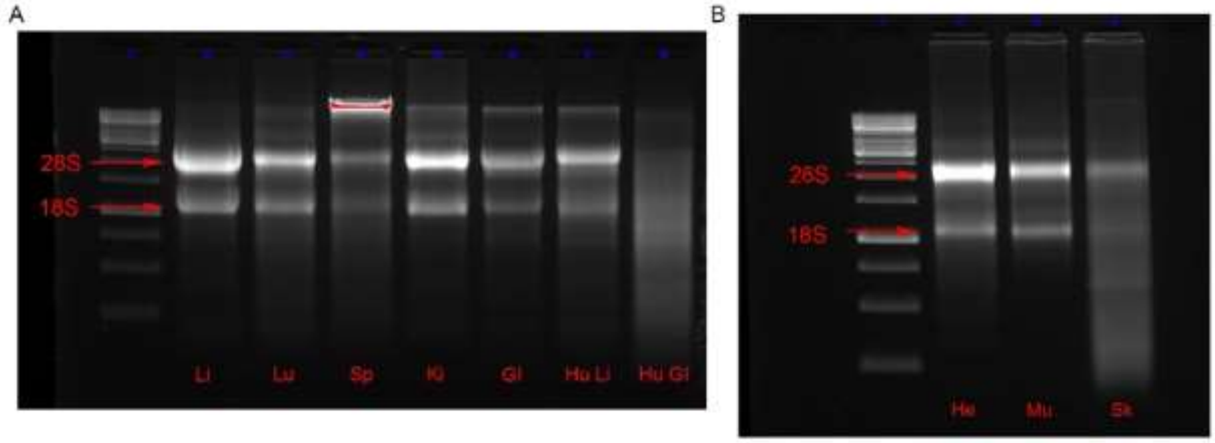

The integrity of RNA extracted from representative mouse and human tissues are shown. (A) RNA extracted from representative mouse liver, lung, spleen, kidney and small intestine are shown in lane 2-6 respectively. (A) RNA extracted from representative human liver and small intestine are shown in lane 7 and 8 respectively. (B) RNA extracted from representative mouse heart, muscle and skin are shown in lane 2-4 respectively.

Table S4. 28S:18S ratio of RNA samples extracted from representative mouse and human tissues

	Mouse<br>28S:18S Ratio	Human<br>28S:18S Ratio
**Liver**	2.51	2.48
**Lung**	2.37	
**Spleen**	1.85	
**Kidney**	2.42	
**GI**	2.96	N/A
**Heart**	3.6	
**Muscle**	2.12	
**Skin**	N/A	

Data are 28S:18S determined from densitometry analysis of RNA samples resolved on 1.2% agarose gel. RNA samples extracted from mouse liver, lung, heart, spleen, muscle, kidney and GI are considered to be intact base on the 28S:18S ratio. The extracted RNA samples from mouse skin showed significant degradation. RNA sample extracted from human liver is considered to be intact while RNA sample extracted from human GI showed significant degradation.

A.

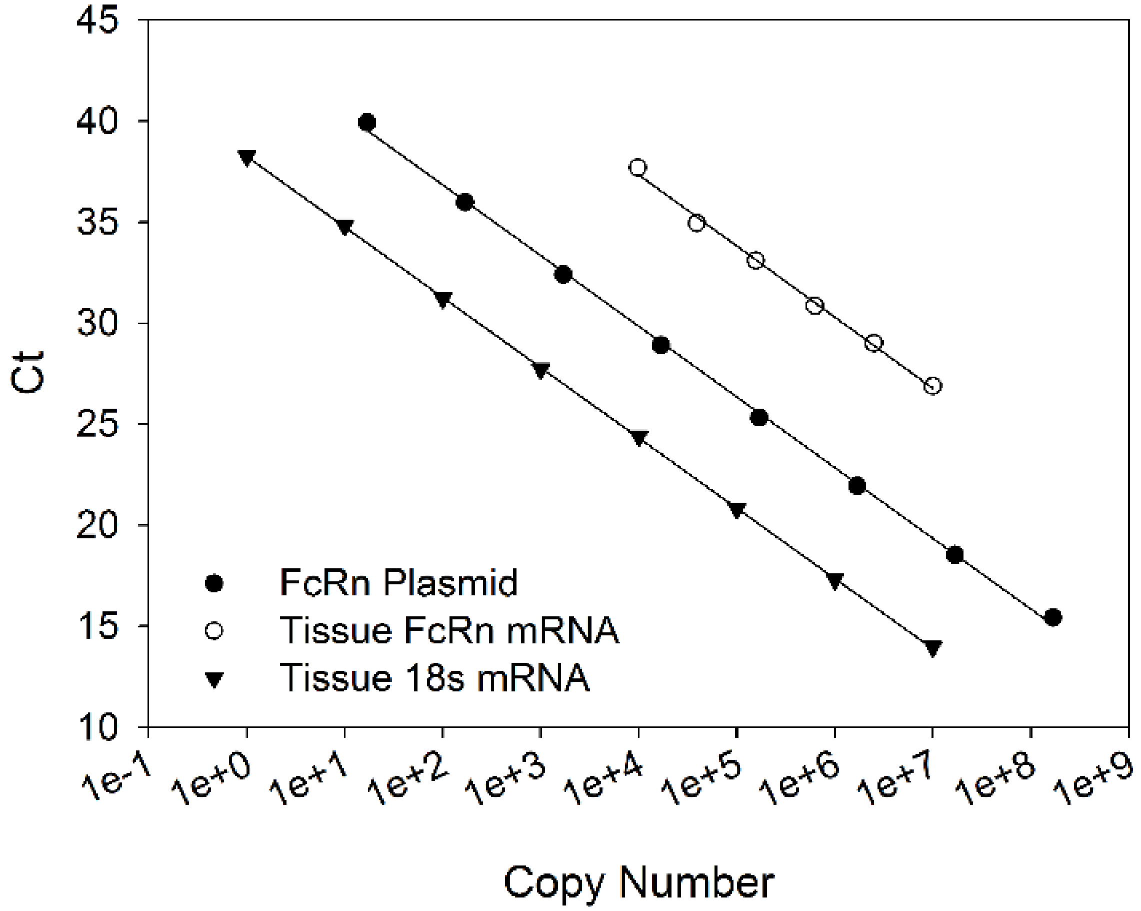

Standard curve constructed from mFcRn plasmid is shown as closed circle, mFcRn standard curve constructed from RNA extracted from mouse tissue is shown as open circle, and 18s standard curve constructed from mouse tissue is shown as closed triangle. The amplification efficiencies were: mFcRn plasmid: 93.2%, tissue mFcRn mRNA: 92.6%, tissue 18s mRNA: 93.9%. The r^2 of standard curve were: mFcRn: 0.9993, tissue mFcRn mRNA: 0.9968, tissue 18s mRNA: 0.9999.

A.

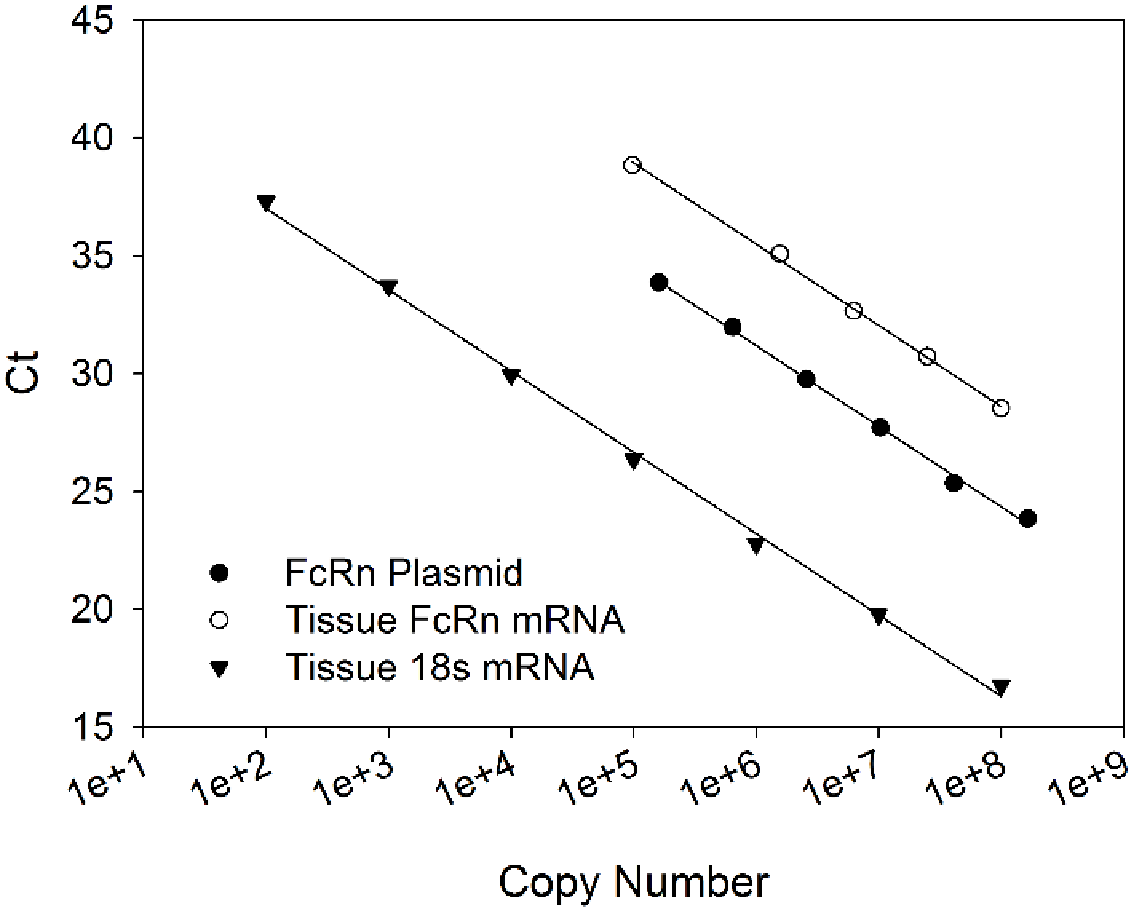

Standard curve constructed from hFcRn plasmid is shown as closed circle, the hFcRn standard curve constructed from RNA extracted from mouse tissue is shown as open circle, and the 18s standard curve constructed from the same mouse tissue is shown as closed triangle. The amplification efficiencies were: hFcRn plasmid: 96.2%, tissue hFcRn mRNA: 95.2%, tissue 18s mRNA: 94.8%. The r^2 of standard curve were: hFcRn: 0.9976, tissue hFcRn mRNA: 0.9985, tissue 18s mRNA: 0.9982.

Western Method Validation

Validation of Western Blot assays was completed for muscle, liver, lung, spleen, small intestine, kidney, heart, and skin tissues of both Swiss Webster and transgenic mice. For quantification of mFcRn in Swiss Webster mice muscle, lung, spleen and heart tissue (Figure S4-7, Table S5-8), we found that use of standards prepared from muscle tissue allowed for accurate recovery and quantification of mFcRn. Quantification of mFcRn in Swiss Webster mice liver, kidney, small intestine and skin tissue required use of standards prepared from these tissues (Figure 8-11, Table 9-12). For quantification of hFcRn in human FcRn transgenic mice muscle and lung tissue (Figure 12 and 13, Table 13 and 14), we found that use of standards prepared from muscle tissue allowed for accurate recovery and quantification of hFcRn.

Quantification of hFcRn in human FcRn transgenic mice spleen, heart, liver, kidney, small intestine and skin tissue required use of standards prepared from these tissues (Figure 14-19, Table 15-20).

Figure S4: Representative validation of quantitative Western Blot method for mFcRn in Swiss Webster muscle tissue.

A

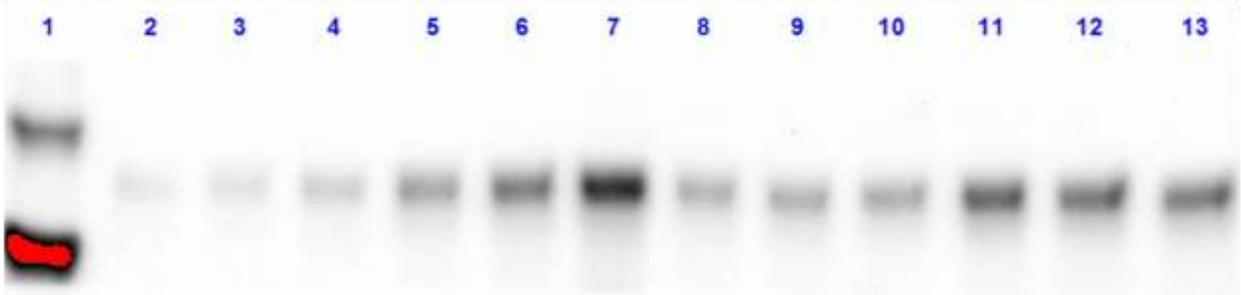

B

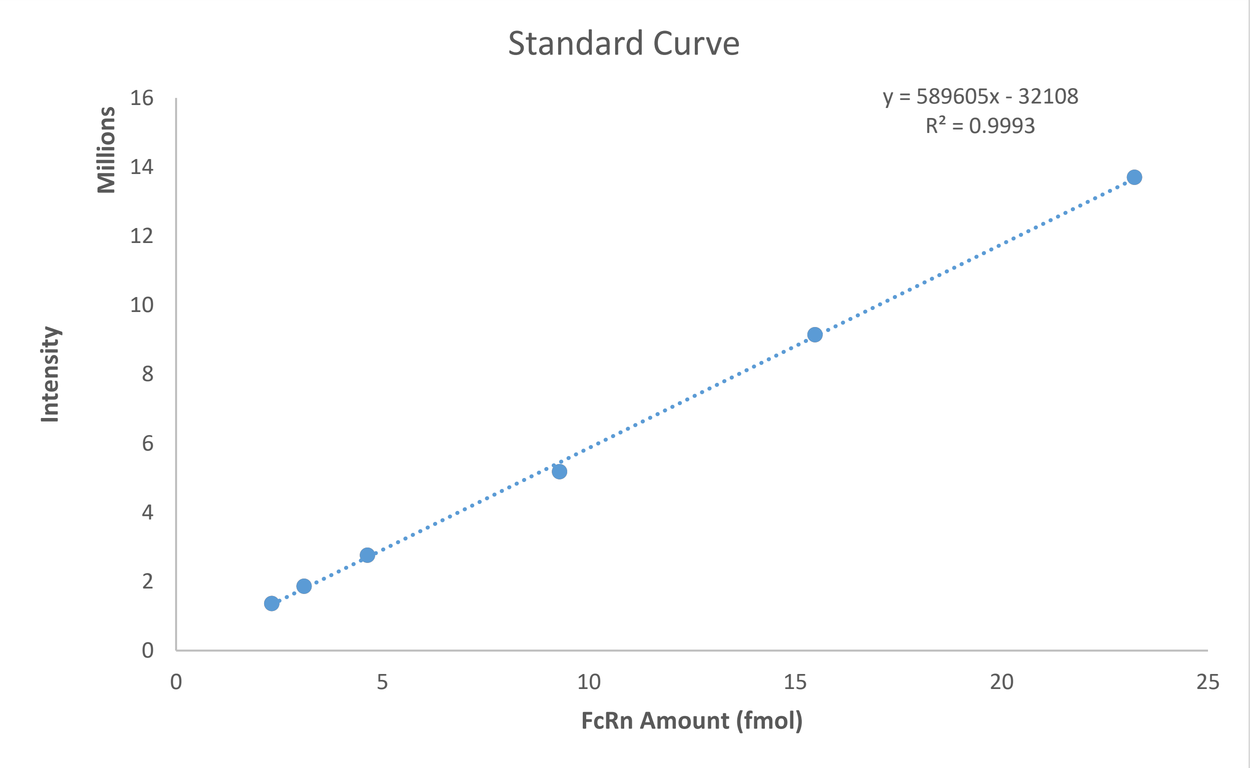

Shown are Western Blot image (A) and corresponding standard curve (B).

Panel A. Western Blot image. Lane 1: 50 kD and 37 kD molecular weight marker. Lane 2-7: standards prepared using transgenic mouse muscle tissue background. Lane 8-10: 3 replicates of QCs at 6.19 fmol prepared from transgenic mouse muscle. Lane 11-13: 3 replicates of QCs at 12.38 fmol prepared from transgenic mouse muscle. Panel B. Standard curve for mFcRn protein in Swiss Webster muscle tissue.

Table S5: Summary of validations of quantitative Western Blot method for SW mouse muscle tissue.

**Summary**		
**QC Amount (fmol)**	Accuracy Average (%)	Inter-assay variability (%CV)
**6.19**	100	6.00
**18.58**	104	6.44

Figure S5: Representative validation of quantitative Western Blot method for mFcRn protein in Swiss Webster lung tissue.

A

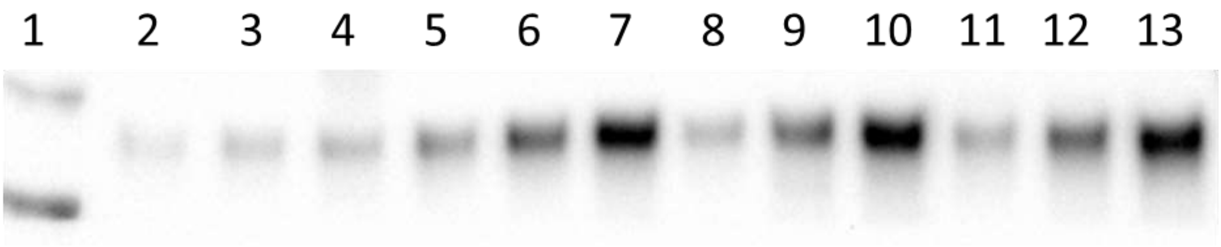

B

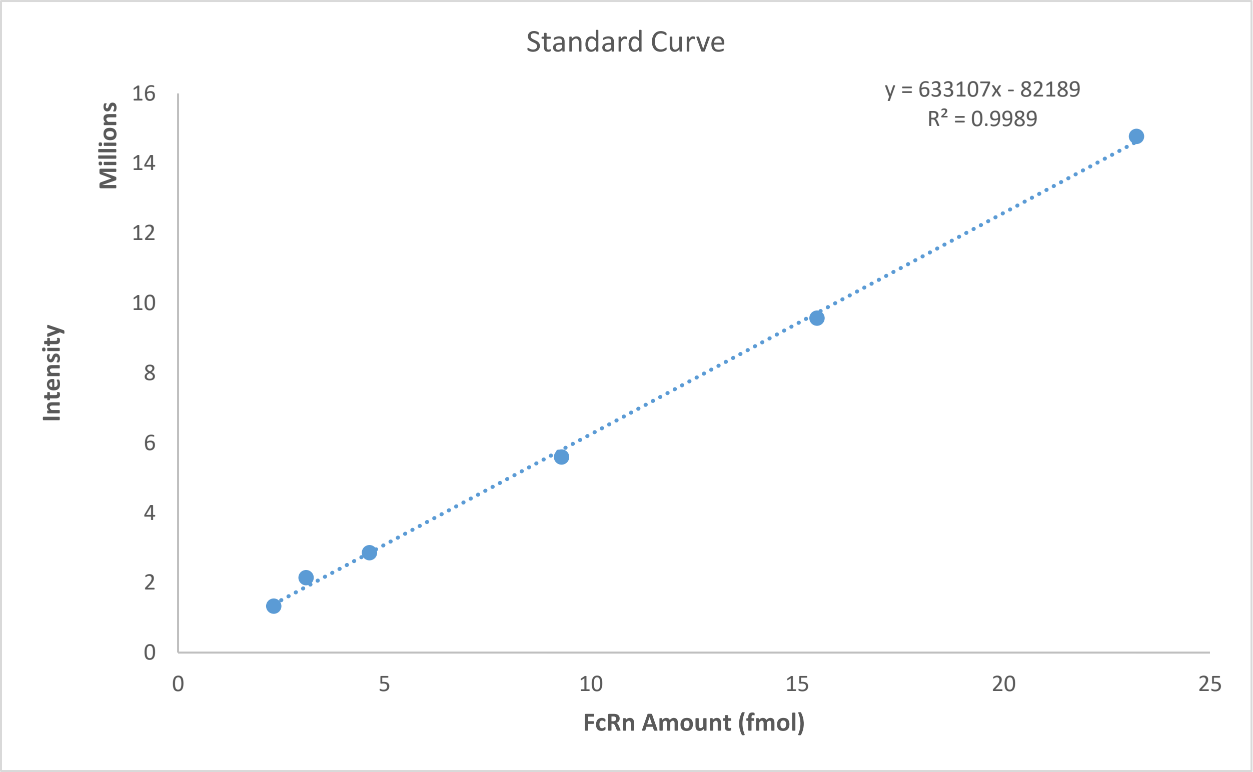

Shown are Western Blot image (A) and corresponding standard curve (B).

Panel A. Western Blot image. Lane 2-7: standards prepared using transgenic mouse muscle tissue background. Lane 8-10: QCs prepared from transgenic mouse muscle tissue at 3.10, 6.19 and 12.38 fmol. Lane 11-13: QCs prepared from transgenic mouse lung tissue at 3.10, 6.19 and 12.38 fmol. Panel B. Standard curve for mFcRn protein in Swiss Webster lung tissue.

Table S6: Summary of validations of quantitative Western Blot method for SW mouse lung tissue.

Summary		
**Tissue Type**	QC Amount (fmol)	Accuracy (%)
**Muscle**	3.10	95.5
**Muscle**	6.19	102
**Muscle**	12.38	107
**Lung**	3.10	101
**Lung**	6.19	94.0
**Lung**	12.38	106

Figure S6: Representative validation of quantitative Western Blot method for mFcRn protein in Swiss Webster spleen tissue.

A

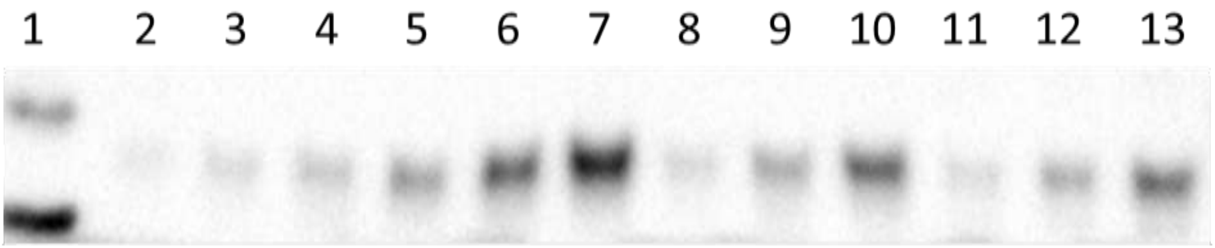

B

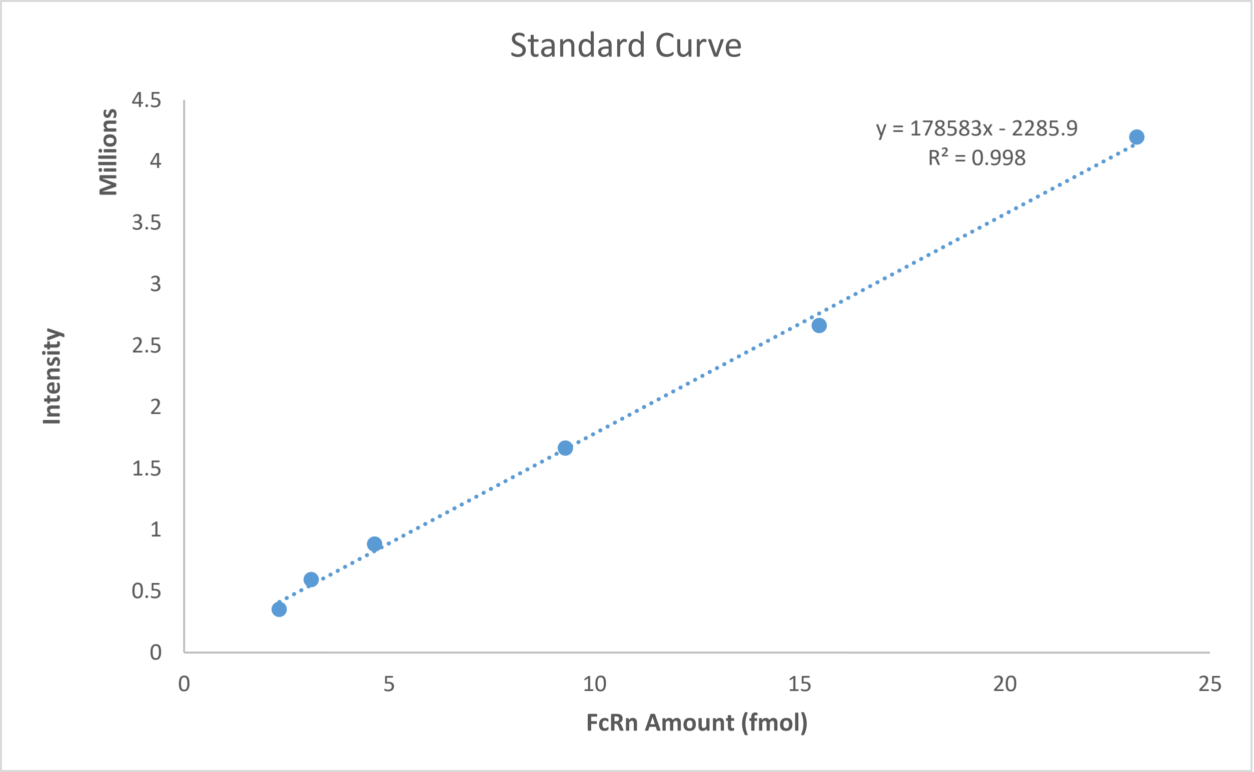

Shown are Western Blot image (A) and corresponding standard curve (B).

Panel A. Western Blot image. Lane 2-7: standards prepared using transgenic mouse muscle tissue background. Lane 8-10: QCs prepared from transgenic mouse muscle tissue at 3.10, 6.19 and 12.38 fmol. Lane 11-13: QCs prepared from transgenic mouse spleen tissue at 3.10, 6.19 and 12.38 fmol. Panel B. Standard curve for mFcRn protein in Swiss Webster spleen tissue.

Table S7: Summary of validations of quantitative Western Blot method for SW mouse spleen tissue.

**Summary**		
**Tissue Type**	QC Amount (fmol)	Accuracy (%)
**Muscle**	3.10	101.8
**Muscle**	6.19	95
**Muscle**	12.38	103
**Spleen**	3.10	99
**Spleen**	6.19	94.8
**Spleen**	12.38	102

Figure S7: Representative validation of quantitative Western Blot method for mFcRn protein in Swiss Webster heart tissue.

A

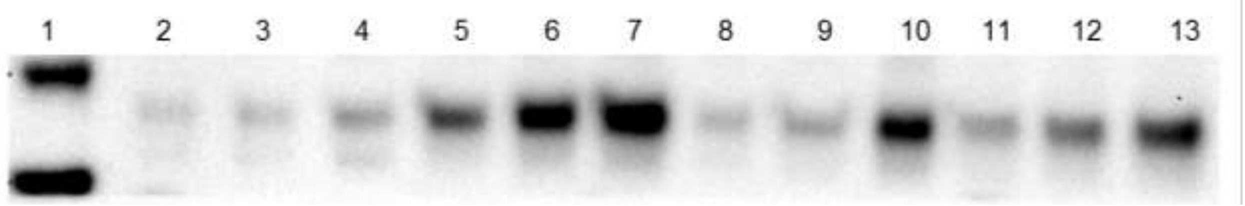

B

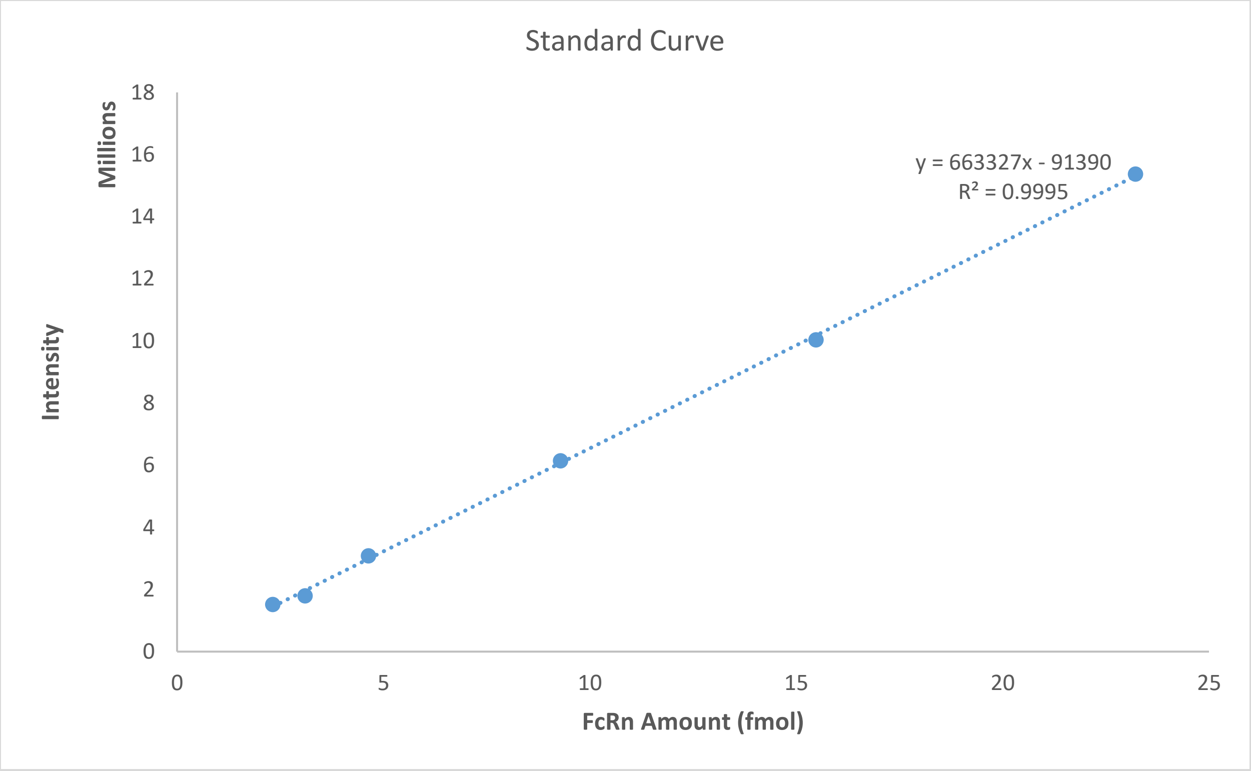

Shown are Western Blot image (A) and corresponding standard curve (B).

Panel A. Western Blot image. Lane 2-7: standards prepared using transgenic mouse muscle tissue background. Lane 8-10: QCs prepared from transgenic mouse muscle tissue at 3.10, 6.19 and 12.38 fmol. Lane 11-13: QCs prepared from transgenic mouse heart tissue at 3.10, 6.19 and 12.38 fmol. Panel B. Standard curve for mFcRn protein in Swiss Webster heart tissue.

Table S8: Summary of validations of quantitative Western Blot method for SW mouse heart tissue.

**Summary**		
**Tissue Type**	QC Amount (fmol)	Accuracy (%)
**Muscle**	3.10	108
**Muscle**	6.19	86.9
**Muscle**	12.38	104
**Heart**	3.10	108
**Heart**	6.19	108
**Heart**	12.38	100

Figure S8: Representative validation of quantitative Western Blot method for mFcRn protein in Swiss Webster kidney tissue.

A

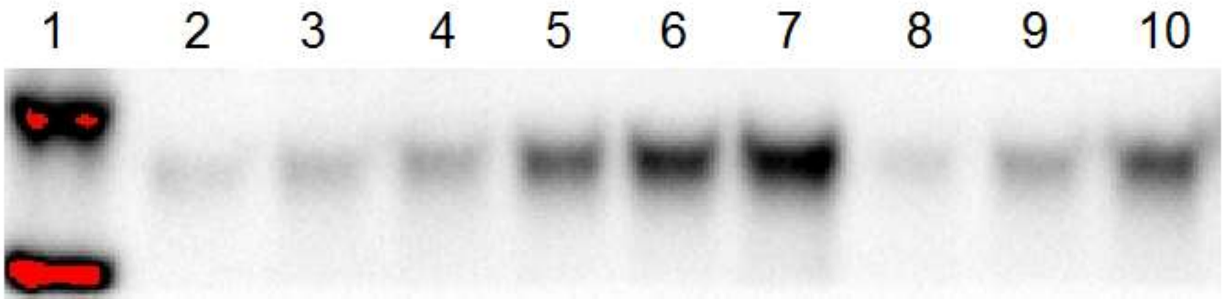

B

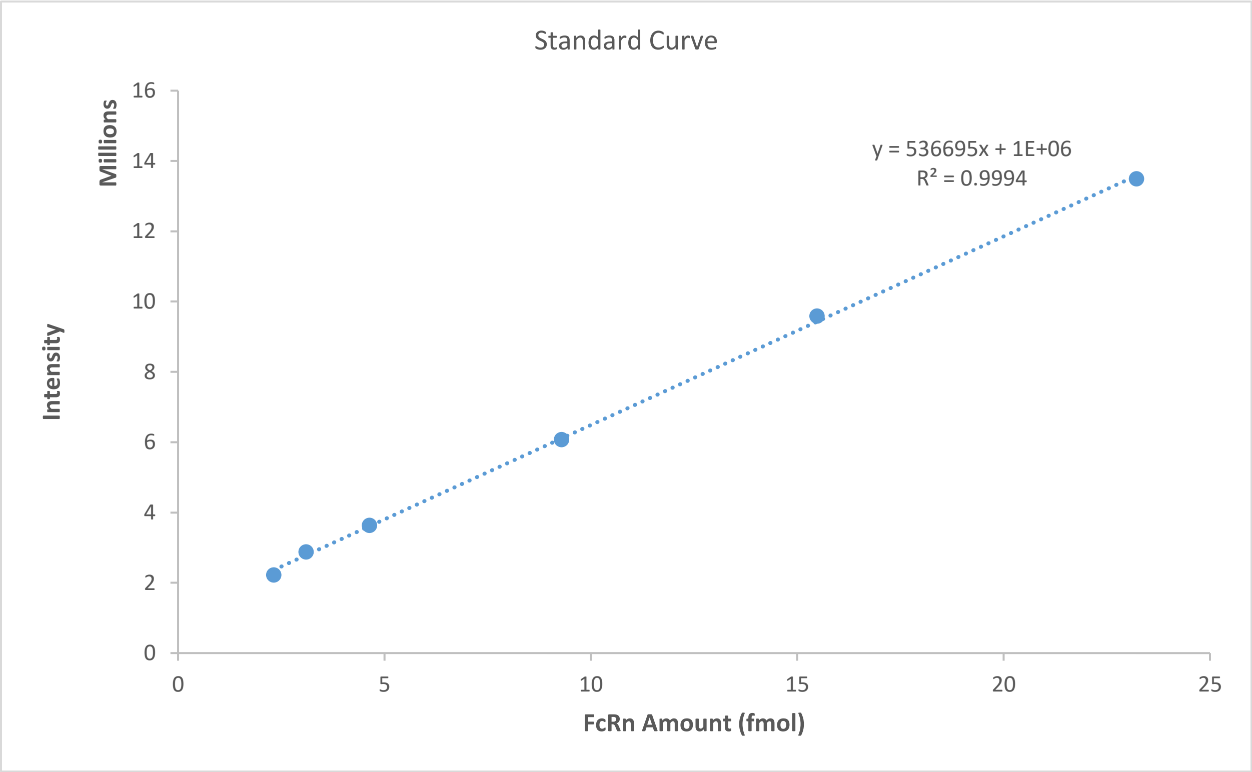

Shown are Western Blot image (A) and corresponding standard curve (B).

Panel A. Western Blot image. Lane 2-7: standards prepared using transgenic mouse kidney tissue background. Lane 8-10: QCs prepared from transgenic mouse kidney tissue at 3.10, 6.19 and 12.38 fmol. Panel B. Standard curve for mFcRn protein in Swiss Webster kidney tissue.

Table S9: Summary of validations of quantitative Western Blot method for SW mouse kidney tissue.

**Summary**		
**QC (fmol)**	Accuracy Avg (%)	Interassay Variabiliy (%CV)
**3.1**	95	9.55
**6.19**	95	4.61
**12.38**	98	1.30

Figure S9: Representative validation of quantitative Western Blot method for mFcRn protein in Swiss Webster small intestine tissue.

A

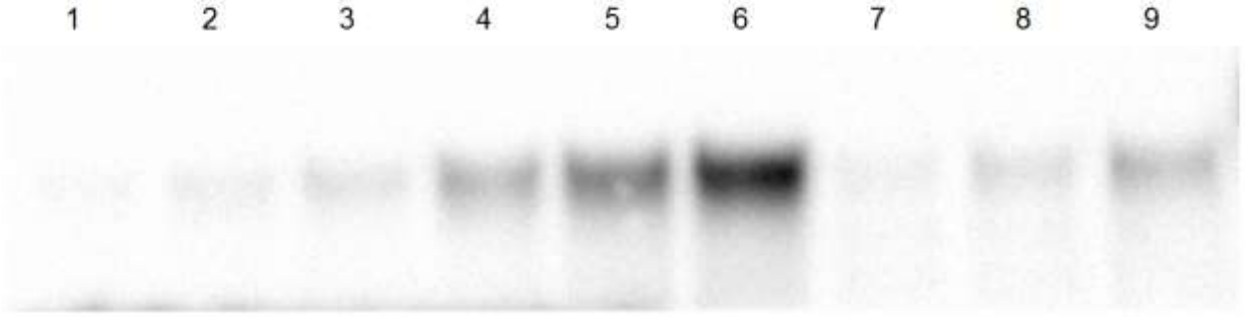

B

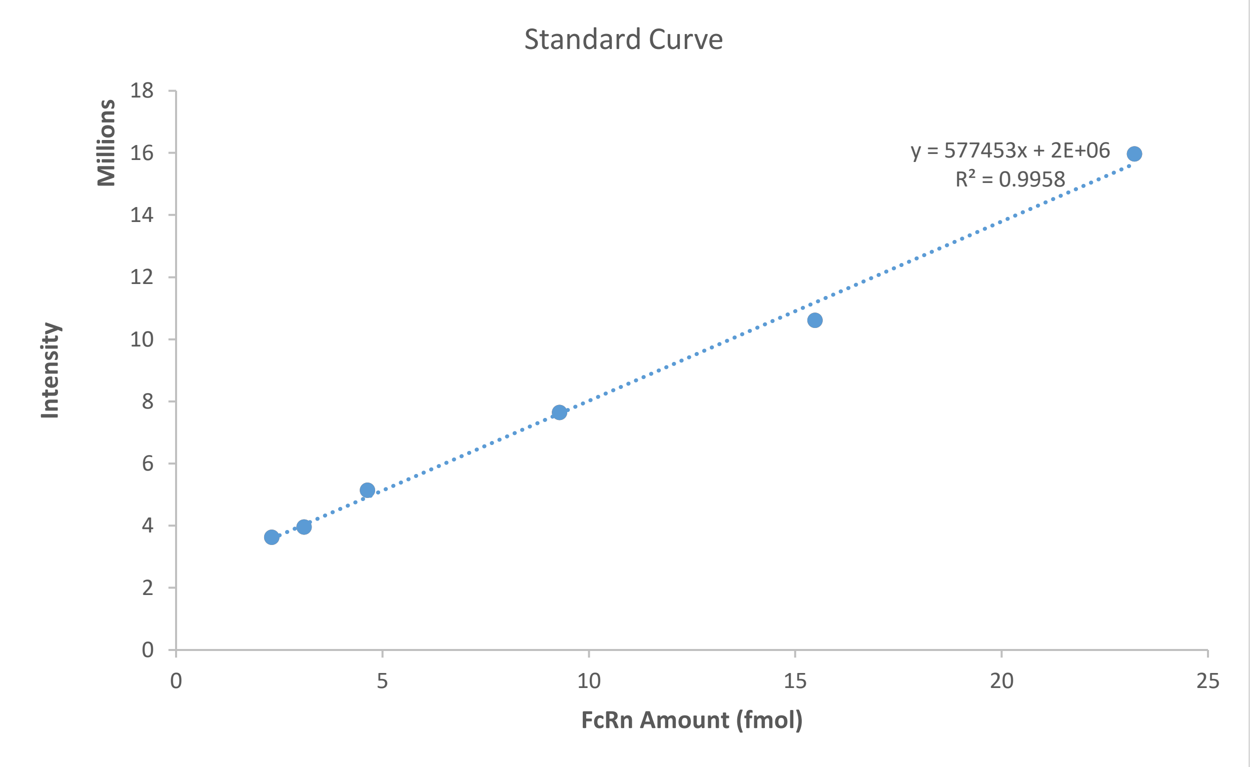

Shown are Western Blot image (A) and corresponding standard curve (B).

Panel A. Western Blot image. Lane 1-6: standards prepared using transgenic mouse small intestine tissue background. Lane 7-9: QCs prepared from transgenic mouse small intestine tissue at 3.10, 6.19 and 12.38 fmol. Panel B. Standard curve for mFcRn protein in Swiss Webster small intestine tissue.

Table S10: Summary of validations of quantitative Western Blot method for SW mouse small intestine tissue.

**Summary**		
**QC (fmol)**	Accuracy Avg (%)	Interassay Variabiliy (%CV)
**3.1**	100	3.59
**6.19**	99	7.59
**12.38**	99	7.87

Figure S10: Representative validation of quantitative Western Blot method for mFcRn protein in Swiss Webster liver tissue.

A

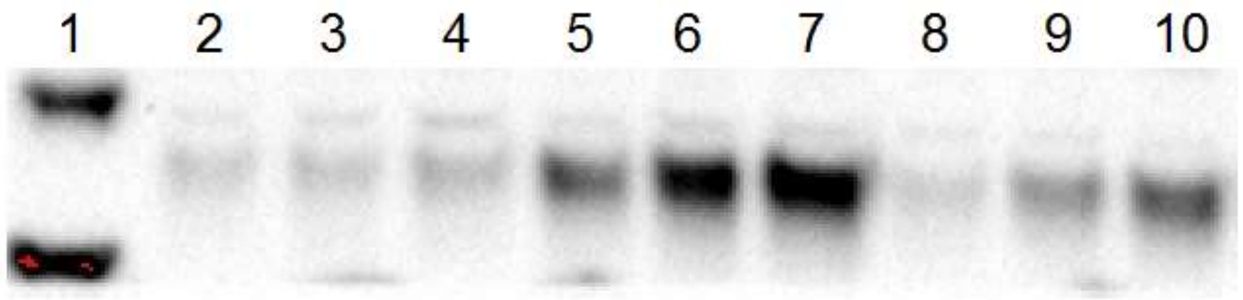

B

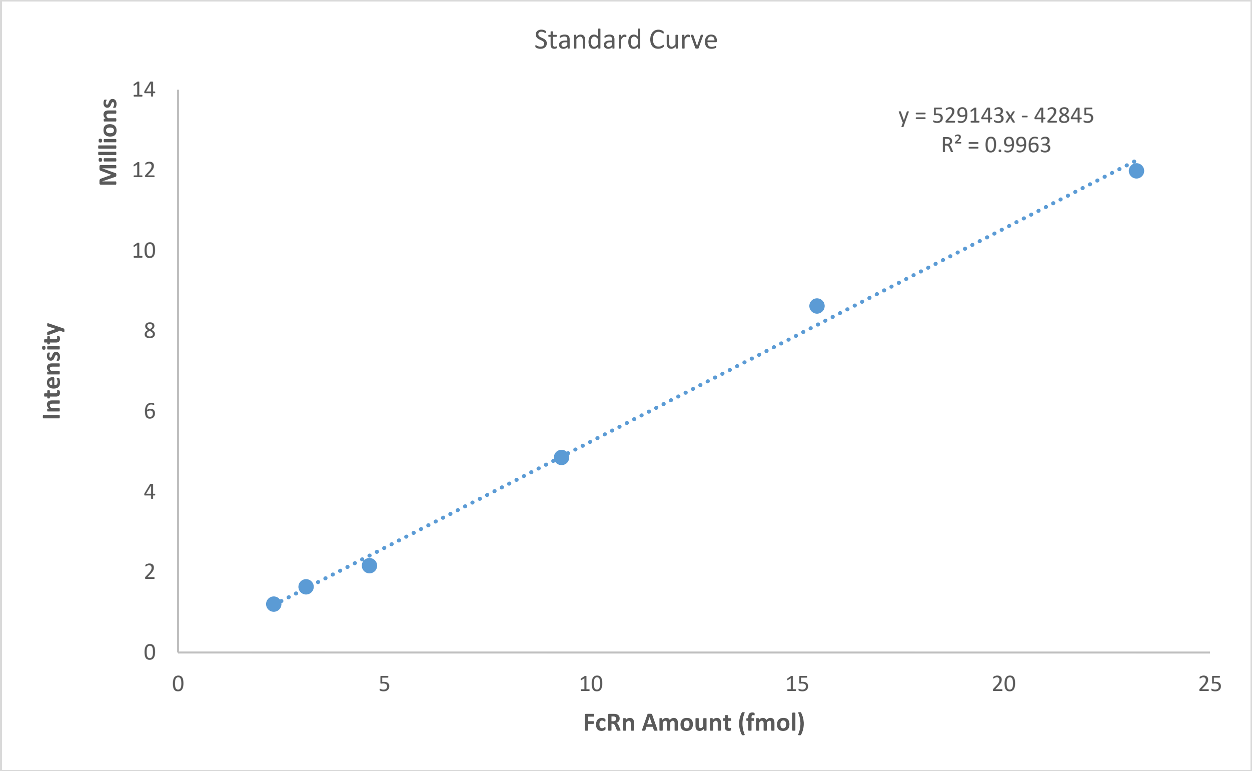

Shown are Western Blot image (A) and corresponding standard curve (B).

Panel A. Western Blot image. Lane 2-7: standards prepared using transgenic mouse liver tissue background. Lane 8-10: QCs prepared from transgenic mouse liver tissue at 3.10, 6.19 and 12.38 fmol. Panel B. Standard curve for mFcRn protein in Swiss Webster liver tissue.

Table S11: Summary of validations of quantitative Western Blot method for SW mouse liver tissue.

**Summary**		
**QC (fmol)**	Accuracy Avg (%)	Interassay Variabiliy (%CV)
**3.1**	110	3.83
**6.19**	99.0	4.87
**12.38**	96.3	3.33

Figure S11: Representative validation of quantitative Western Blot method for mFcRn protein in Swiss Webster skin tissue.

A

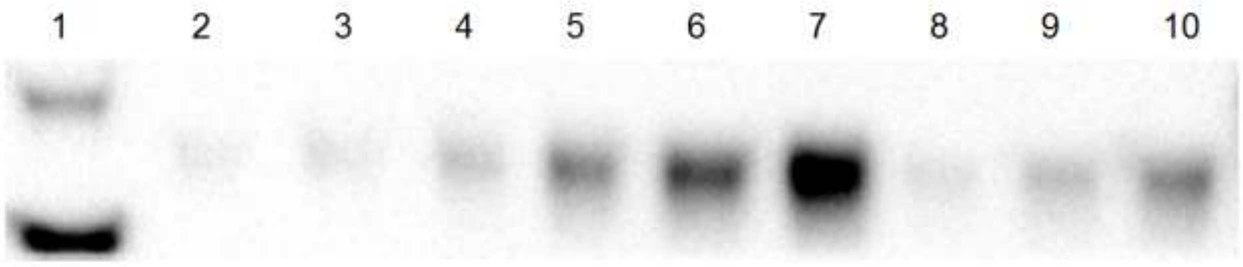

B

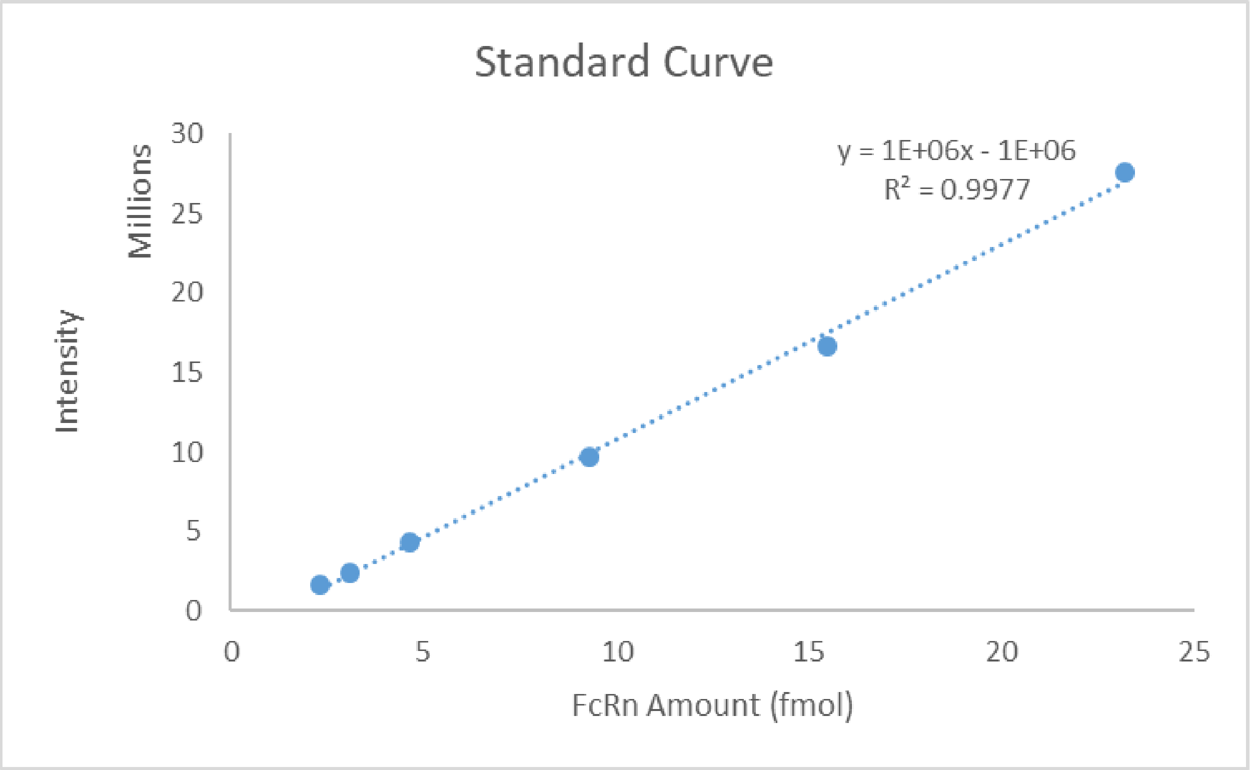

Shown are Western Blot image (A) and corresponding standard curve (B).

Panel A. Western Blot image. Lane 2-7: standards prepared using transgenic mouse skin tissue background. Lane 8-10: QCs prepared from transgenic mouse skin tissue at 3.10, 6.19 and 12.38 fmol. Panel B. Standard curve for mFcRn protein in Swiss Webster skin tissue.

Table S12: Summary of validations of quantitative Western Blot method for SW mouse skin tissue

**Summary**		
**QC Amount (fmol)**	Accuracy Average (%)	Inter-assay variability (%CV)
**3.1**	110	3.83
**6.19**	99	4.87
**12.38**	96	3.33

Figure S12: Representative validation of quantitative Western Blot method for hFcRn protein in transgenic mouse muscle tissue.

A

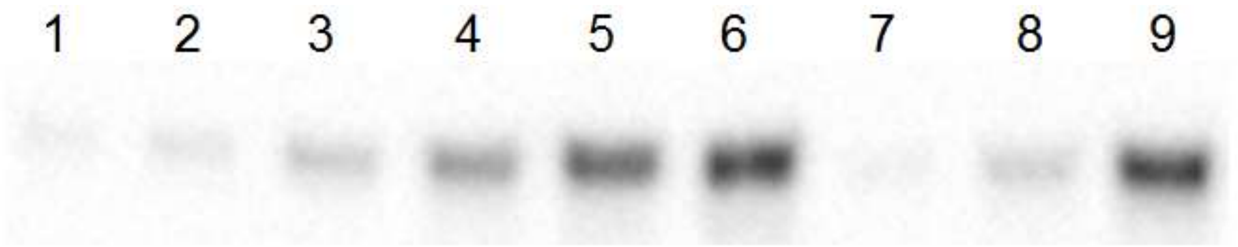

B

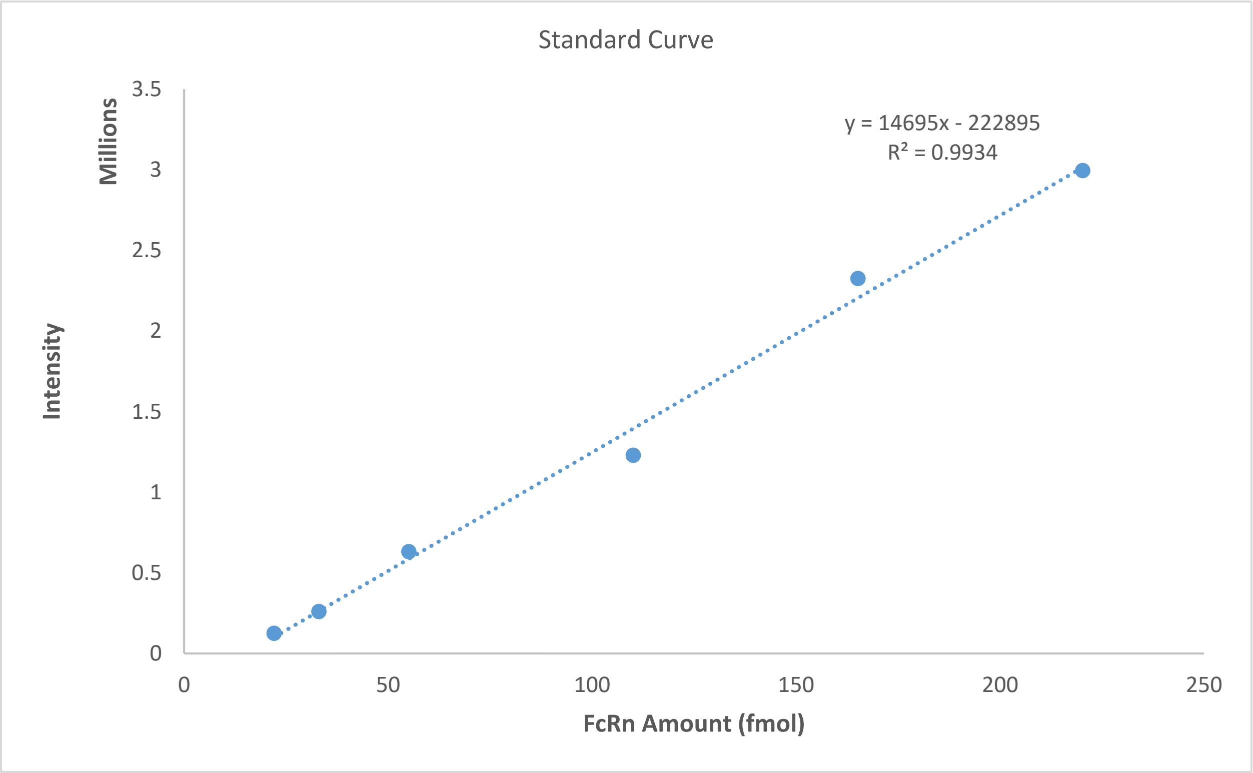

Shown are Western Blot image (A) and corresponding standard curve (B).

Panel A. Western Blot image. Lane 1-6: standards prepared using C57BL6 mouse muscle tissue background. Lane 7-9: QCs prepared from C57BL6 mouse muscle tissue at 27.5, 88.1 and 176 fmol. Panel B. Standard curve for hFcRn protein in transgenic mouse muscle tissue.

Table S13: Summary of validations of quantitative Western Blot method for transgenic mouse muscle tissue.

**Summary**		
**QC Amount (fmol)**	Accuracy Average (%)	Inter-assay variability (%CV)
**27.5**	93.0	4.60
**88.1**	80.8	0.73
**176**	106	4.37

Figure S13: Representative validation of quantitative Western Blot method for hFcRn protein in transgenic mouse lung tissue.

A

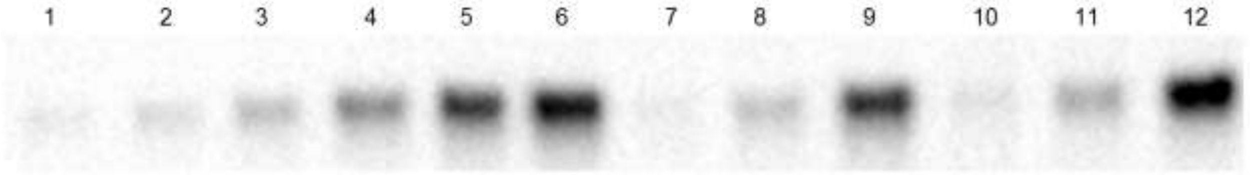

B

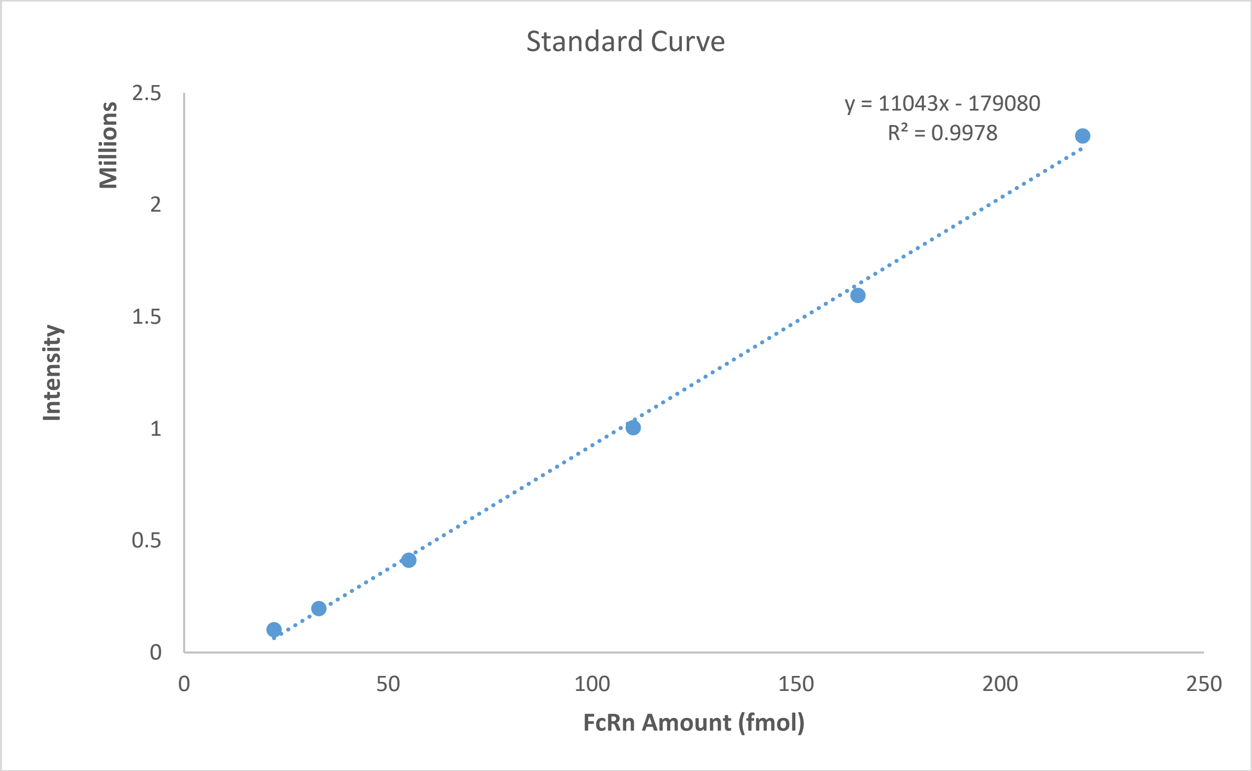

Shown are Western Blot image (A) and corresponding standard curve (B).

Panel A. Western Blot image. Lane 1-6: standards prepared using C57BL6 mouse muscle tissue background. Lane 7-9: QCs prepared from C57BL6 mouse muscle tissue at 27.5, 88.1 and 176 fmol. Lane 10-12: QCs prepared from C57BL6 mouse lung tissue at 27.5, 88.1 and 176 fmol. Panel B. Standard curve for hFcRn protein in transgenic mouse lung tissue.

Table S14: Summary of validations of quantitative Western Blot method for transgenic mouse lung tissue.

**Summary**		
**Tissue Type**	QC Amount (fmol)	Accuracy (%)
**Muscle**	27.5	100
**Muscle**	88.1	82.6
**Muscle**	176.2	113
**Lung**	27.5	97.9
**Lung**	88.1	84.6
**Lung**	176.2	118

Figure S14: Representative validation of quantitative Western Blot method for hFcRn protein in transgenic mouse spleen tissue.

A

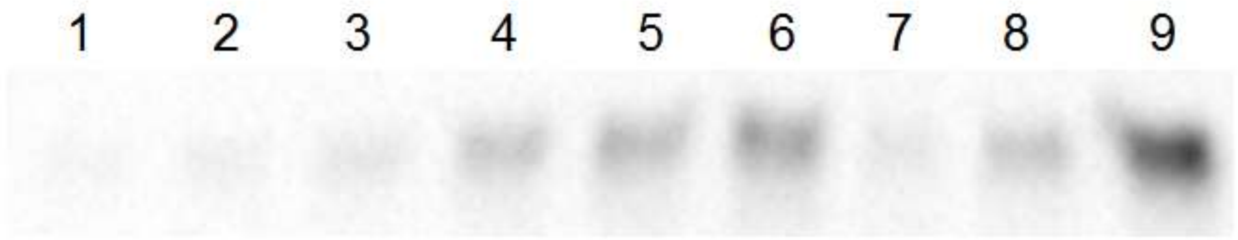

B

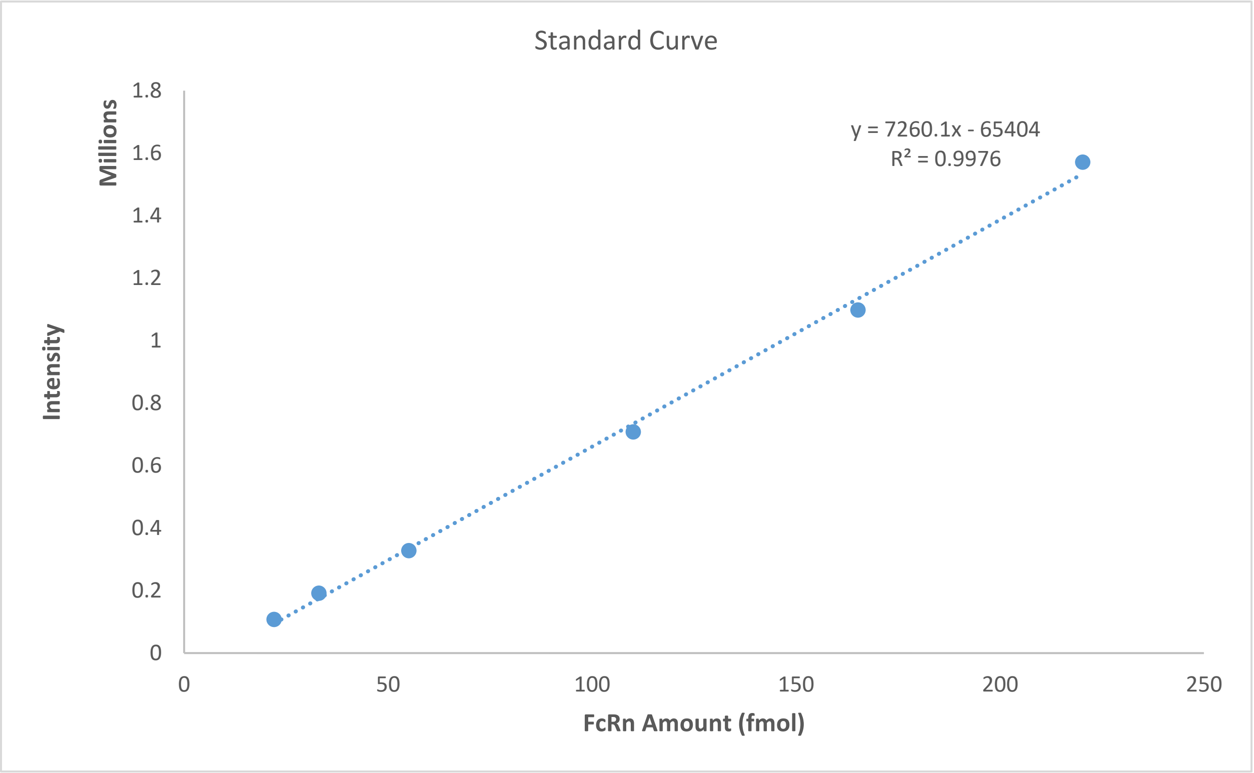

Shown are Western Blot image (A) and corresponding standard curve (B).

Panel A. Western Blot image. Lane 1-6: standards prepared using C57BL6 mouse spleen tissue background. Lane 7-9: QCs prepared from C57BL6 mouse spleen tissue at 27.5, 88.1 and 176 fmol. Panel B. Standard curve for hFcRn protein in transgenic mouse spleen tissue.

Table S15: Summary of validations of quantitative Western Blot method for transgenic mouse spleen tissue.

**Summary**		
**QC (fmol)**	Accuracy Avg (%)	Interassay Variabiliy (%CV)
**27.5**	110	2.48
**88.1**	94.0	5.12
**176**	119	0.99

Figure S15: Representative validation of quantitative Western Blot method for hFcRn protein in transgenic mouse heart tissue.

A

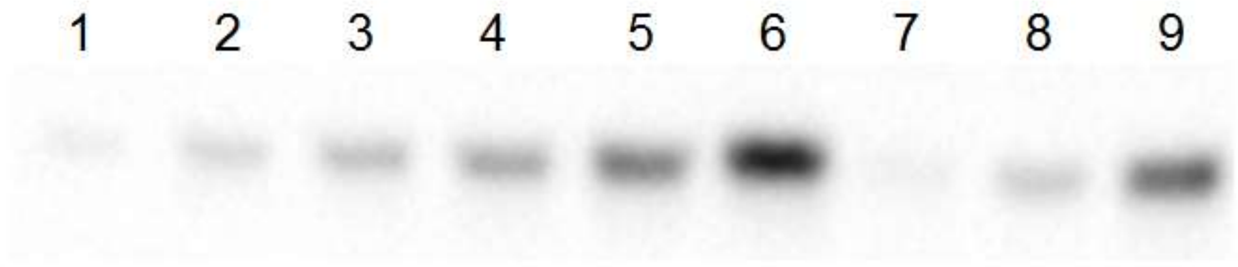

B

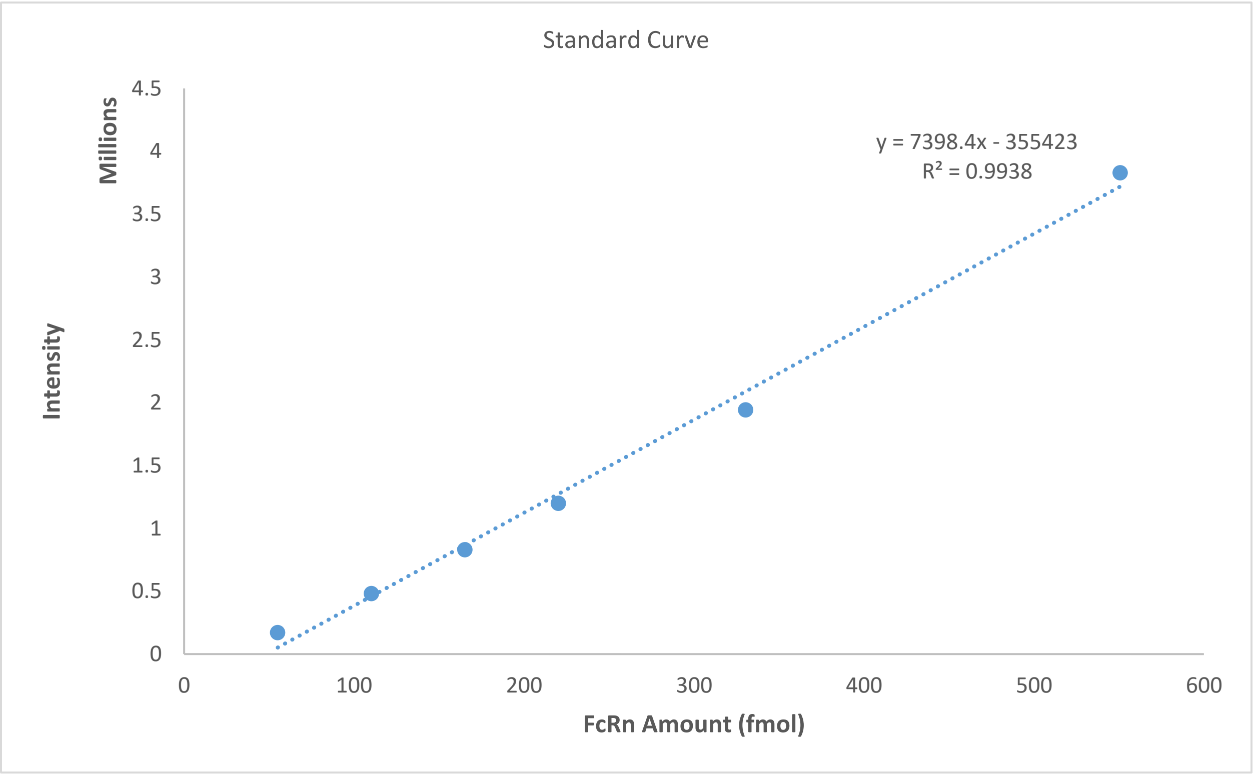

Shown are Western Blot image (A) and corresponding standard curve (B).

Panel A. Western Blot image. Lane 1-6: standards prepared using C57BL6 mouse heart tissue background. Lane 7-9: QCs prepared from C57BL6 mouse heart tissue at 77.1, 198 and 396 fmol. Panel B. Standard curve for hFcRn protein in transgenic mouse heart tissue.

Table S16: Summary of validations of quantitative Western Blot method for transgenic mouse heart tissue.

**Summary**		
**QC (fmol)**	Accuracy Avg (%)	Interassay Variabiliy (%CV)
**77.1**	89.3	11.3
**198**	84.1	2.97
**396**	103	8.80

Figure S16: Representative validation of quantitative Western Blot method for hFcRn protein in transgenic mouse kidney tissue.

A

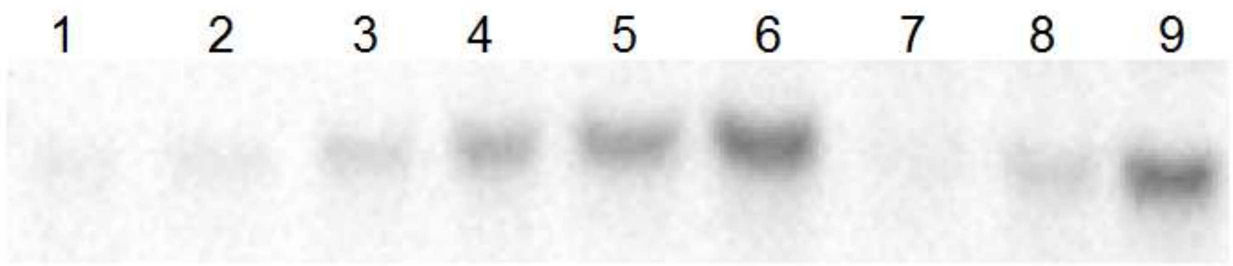

B

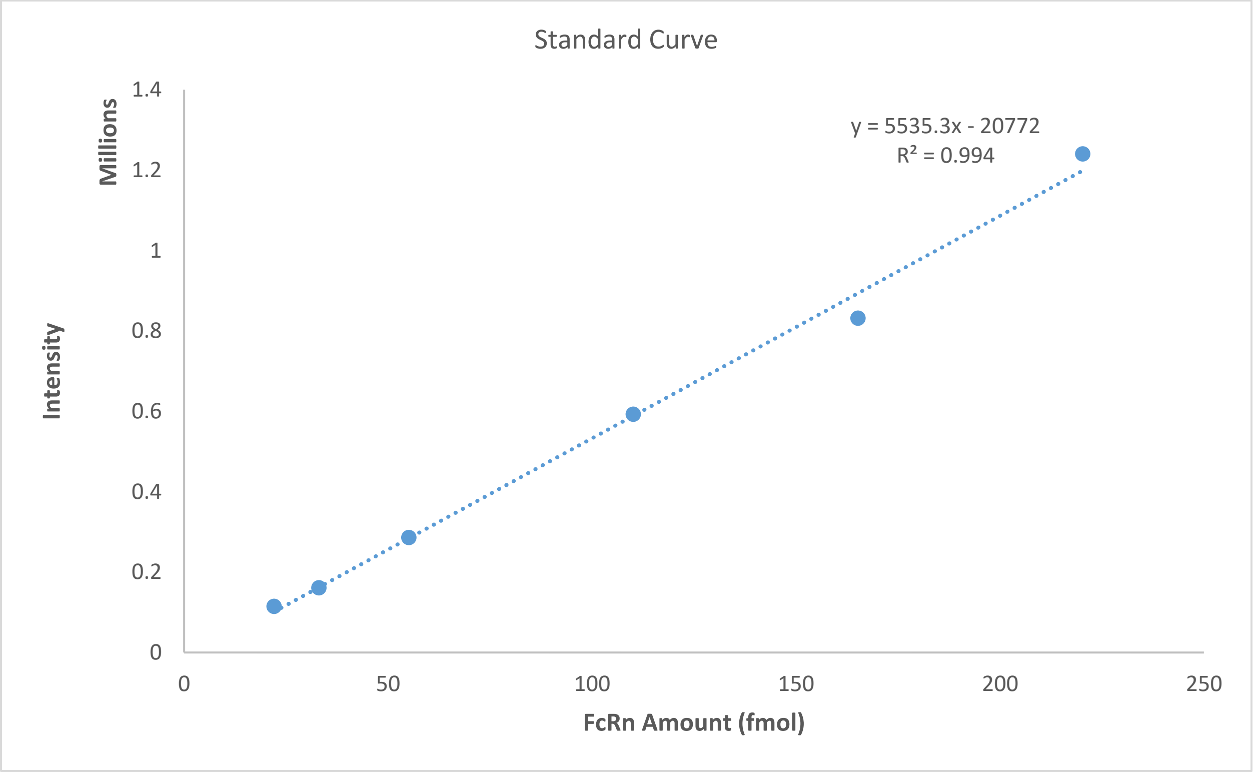

Shown are Western Blot image (A) and corresponding standard curve (B).

Panel A. Western Blot image. Lane 1-6: standards prepared using C57BL6 mouse kidney tissue background. Lane 7-9: QCs prepared from C57BL6 mouse kidney tissue at 27.5, 88.1 and 176 fmol. Panel B. Standard curve for hFcRn protein in transgenic mouse kidney tissue.

Table S17: Summary of validations of quantitative Western Blot method for transgenic mouse kidney tissue.

**Summary**		
**QC (fmol)**	Accuracy Avg (%)	Interassay Variabiliy (%CV)
**27.5**	84.7	0.954
**88.1**	81.8	2.09
**176**	103	6.16

Figure S17: Representative validation of quantitative Western Blot method for hFcRn protein in transgenic mouse small intestine tissue.

A

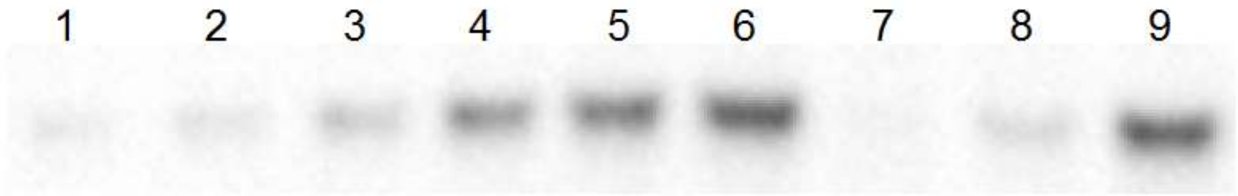

B

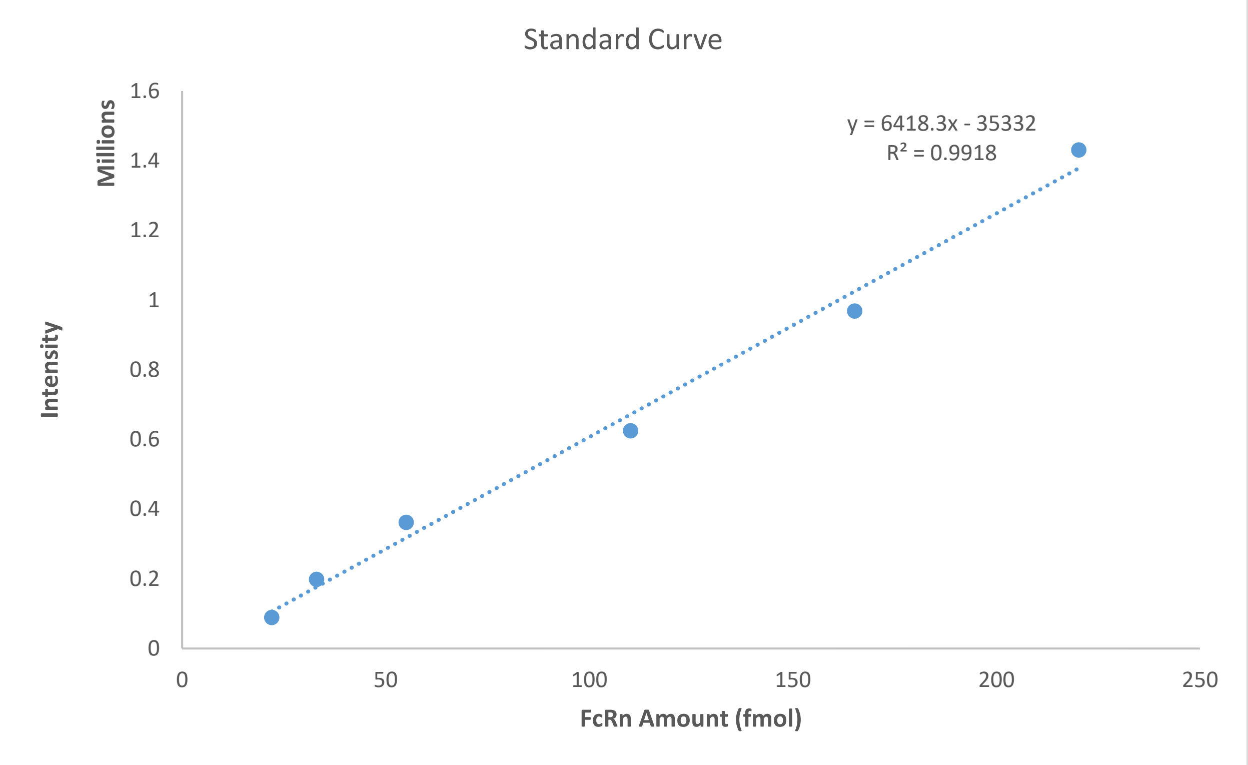

Shown are Western Blot image (A) and corresponding standard curve (B).

Panel A. Western Blot image. Lane 1-6: standards prepared using C57BL6 mouse small intestine tissue background. Lane 7-9: QCs prepared from C57BL6 mouse small intestine tissue at 27.5, 88.1 and 176 fmol. Panel B. Standard curve for hFcRn protein in transgenic mouse small intestine tissue.

Table S18: Summary of validations of quantitative Western Blot method for transgenic mouse small intestine tissue.

**Summary**		
**QC (fmol)**	Accuracy Avg (%)	Interassay Variabiliy (%CV)
**27.5**	85.9	7.32
**88.1**	80.7	0.508
**176**	112	9.65

Figure S18: Representative validation of quantitative Western Blot method for hFcRn protein in transgenic mouse liver tissue.

A

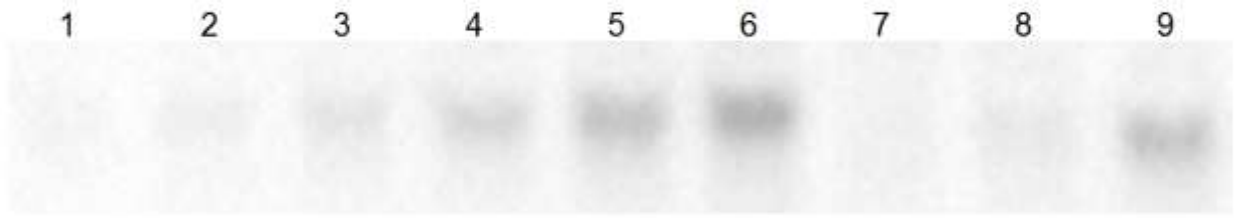

B

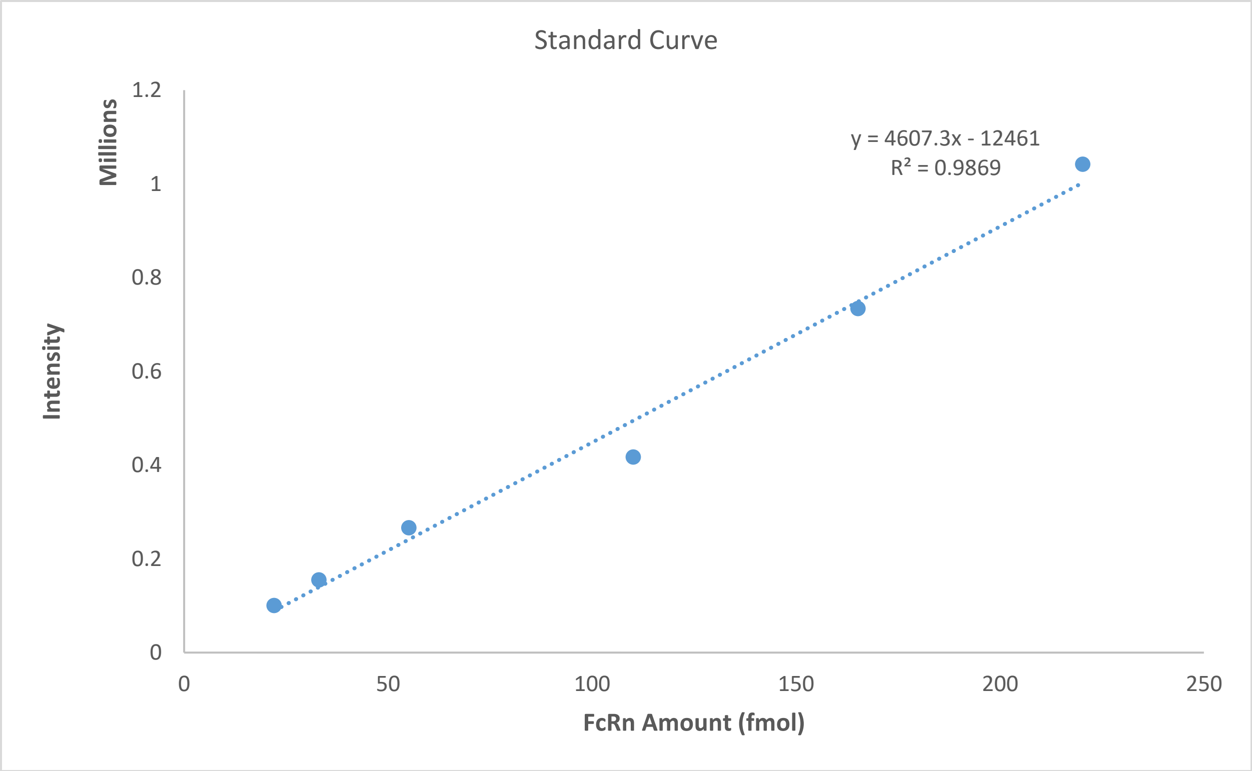

Shown are Western Blot image (A) and corresponding standard curve (B).

Panel A. Western Blot image. Lane 1-6: standards prepared using C57BL6 mouse liver tissue background. Lane 7-9: QCs prepared from C57BL6 mouse liver tissue at 27.5, 88.1 and 176 fmol. Panel B. Standard curve for hFcRn protein in transgenic mouse liver tissue.

Table S19: Summary of validations of quantitative Western Blot method for transgenic mouse liver tissue.

**Summary**		
**QC (fmol)**	Accuracy Avg (%)	Interassay Variabiliy (%CV)
**27.5**	85.4	2.08
**88.1**	80.5	0.870
**176**	100	8.10

Figure S19: Representative validation of quantitative Western Blot method for hFcRn protein in transgenic mouse skin tissue.

A

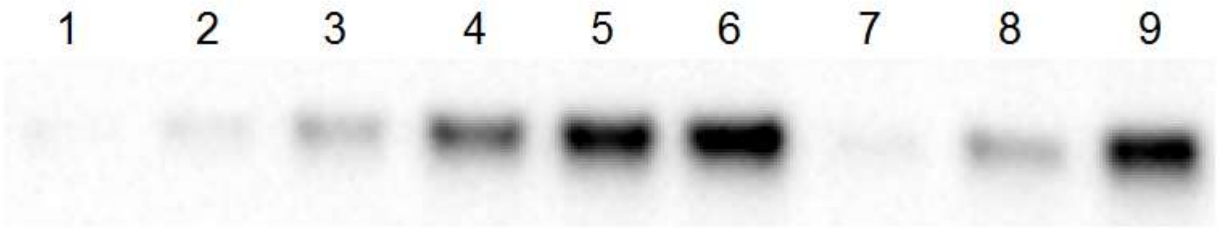

B

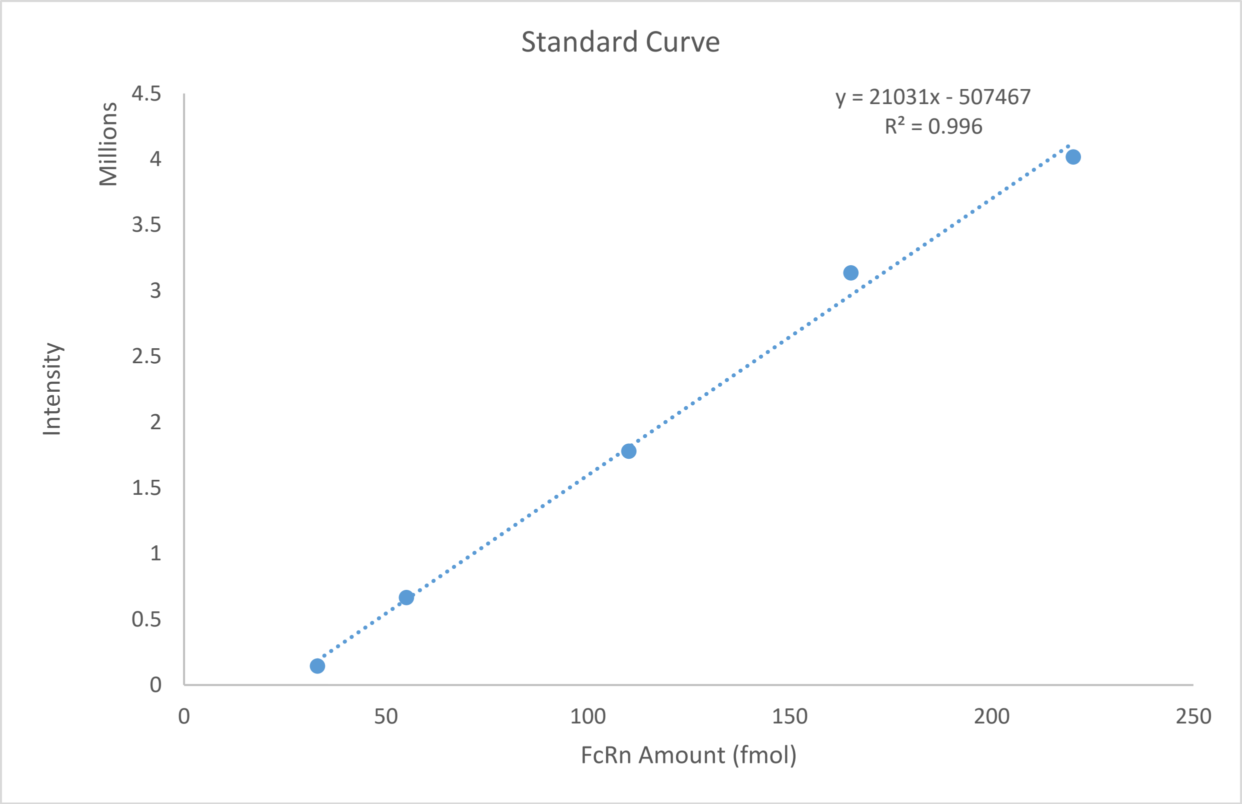

Shown are Western Blot image (A) and corresponding standard curve (B).

Panel A. Western Blot image. Lane 1-6: standards prepared using C57BL6 mouse skin tissue background. Lane 7-9: QCs prepared from C57BL6 mouse skin tissue at 44.05, 88.11 and 176.21 fmol. Panel B. Standard curve for hFcRn protein in transgenic mouse skin tissue.

Table S20: Summary of validations of quantitative Western Blot method for transgenic mouse skin tissue.

**Summary**		
**QC (fmol)**	Accuracy Avg (%)	Interassay Variabiliy (%CV)
**44.05**	99.3	14.21
**88.11**	83.1	1.27
**176.21**	97.4	5.66
